# Use of mathematical modelling to assess respiratory syncytial virus epidemiology and interventions: a literature review

**DOI:** 10.1007/s00285-021-01706-y

**Published:** 2022-02-26

**Authors:** John C. Lang

**Affiliations:** Biostatistics and Research Decision Sciences (BARDS), Merck & Co., Inc., Kennilworth, NJ USA

**Keywords:** Respiratory syncytial virus, Infectious disease model, Dynamic transmission model, Vaccination, Immunoprophylaxis, 92D30, 37N25

## Abstract

Respiratory syncytial virus (RSV) is a leading cause of acute lower respiratory tract infection worldwide, resulting in approximately sixty thousand annual hospitalizations of< 5-year-olds in the United States alone and three million annual hospitalizations globally. The development of over 40 vaccines and immunoprophylactic interventions targeting RSV has the potential to significantly reduce the disease burden from RSV infection in the near future. In the context of RSV, a highly contagious pathogen, dynamic transmission models (DTMs) are valuable tools in the evaluation and comparison of the effectiveness of different interventions. This review, the first of its kind for RSV DTMs, provides a valuable foundation for future modelling efforts and highlights important gaps in our understanding of RSV epidemics. Specifically, we have searched the literature using Web of Science, Scopus, Embase, and PubMed to identify all published manuscripts reporting the development of DTMs focused on the population transmission of RSV. We reviewed the resulting studies and summarized the structure, parameterization, and results of the models developed therein. We anticipate that future RSV DTMs, combined with cost-effectiveness evaluations, will play a significant role in shaping decision making in the development and implementation of intervention programs.

**Supplementary Information:**

The online version contains supplementary material available at 10.1007/s00285-021-01706-y.

## Introduction

Respiratory syncytial virus (RSV), a highly contagious disease, has increasingly been recognized as a leading cause of acute lower respiratory tract infection worldwide (Nair et al. 2010, 2013). The overwhelming majority of individuals are infected by their second year of life (Glezen et al. 1986; Henderson et al. 1979). Severe disease is most common in young infants ($< 6$-month-olds), with incidence decreasing rapidly with age (Hall et al. 2009, 2013; Rha et al. 2020). Globally, it is estimated that RSV is responsible for approximately three million hospitalizations annually in children ($< 5$-year-olds) (Nair et al. 2010, 2013). Lifelong reinfections with RSV are common and, although healthy older children (5–17-year-olds) and adults (18–64-year-olds) are less likely to develop severe disease (Hall et al. 2001), severe disease is more common in older adults ($\ge 65$-year-olds), institutionalized individuals, and immunocompromised individuals (Falsey et al. 2005; Widmer et al. 2012, 2014). Respiratory syncytial virus epidemics exhibit rich dynamics that vary geographically and climatically; both annual peaks, and biennial alternating high- and low-peaks, have been observed in RSV epidemics (Bloom-Feshbach et al. 2013; Li et al. 2019).

At present there is only one immunoprophylaxis, the monoclonal antibody palivizumab, that is recommended for the prevention of RSV disease; however, due to its high expense and limited effectiveness, recommendations are generally limited to high-risk patients, i.e., very premature infants, infants with chronic lung disease (CLD), or infants with congenital heart disease (CHD) (Committee on Infectious Diseases 2014; Gutfraind et al. 2015). Nevertheless, development of RSV immunoprophylactic interventions are proceeding rapidly, with over 40 RSV vaccines or immunoprophylactic interventions currently under development (Higgins et al. 2016; PATH 2020).

Mathematical models play an important role in many aspects of epidemiological research (Chubb and Jacobsen 2010). For example, the US Centers for Disease Control and Prevention has recently developed a static model that can be applied to evaluate the number of medically attended RSV infections subject to various interventions (Rainisch et al. 2020). Whereas static models are effective at estimating the direct effects of immunoprophylactic interventions, they are ill-suited to the study of indirect effects or herd immunity effects, which are frequently significant for infectious diseases (Pitman et al. 2012). Thus, in anticipation of the availability of multiple immunoprophylactic options for RSV there has been increasing interest in the development of dynamic transmission models (DTMs) that are fully capable of representing complex interactions between virus, environment, population, and immunoprophylactic interventions. As with static models, DTMs can be integrated into cost effectiveness analyses to aid public policy decision making with respect to the control of RSV.

The principal aim of this literature review is to provide an overview of RSV DTMs as a resource for future RSV dynamic modelling efforts. We proceed in four parts. First, we outline RSV DTM structures. Second, we summarize data sources used for model calibration and common parameter values determined through model parameterization. Third, we present the main findings of RSV modelling papers. Finally, we identify key areas for future modelling research and discuss how mathematical modelling can contribute to public health decision making.

We note that, unlike a systemic review (Munn et al. 2018), this literature review does not consider a specific research question. Because model structure and modelling technique employed are often a function of both data availability (e.g., for model calibration and parameterization) and the specific research question being investigated, comparison between different RSV DTMs is not always well defined. As such, the presentation of a broad overview of RSV DTMs has been prioritized over analyses comparing and contrasting model structures or modelling techniques. In other words, whereas some general comparisons between RSV DTMs are made in a general context, complex analyses comparing and contrasting RSV DTMs are beyond the scope of this literature review and are left as future work. Similarly, whereas there exists extensive research on animal transmission (Greenhalgh et al. 2000; Greenhalgh and Griffiths 2009; Smith et al. 2014) and within-host (González-Parra and Dobrovolny 2018, 2019; Khan and Dobrovolny 2021) RSV dynamics, the complex nature of these topics preclude them from the scope of this review.

## Search strategy and results

### Search strategy and selection criteria

Studies for this review were identified through searches of Web of Science (Clarivate Analytics 2020), Embase (Elsevier 2020a), Scopus (Elsevier 2020b), and PubMed (National Center for Biotechnology Information 2020), by use of terms (a) “respiratory syncytial virus”, “human respiratory syncytial virus”, “rsv”, or “hrsv”, and (b) “mathematical model”, “dynamic transmission model”, “dynamic model”, “transmission model”, “epidemic model”, “compartment model”, or “compartmental model”. For Embase and PubMed searches we add corresponding Emtree (“respiratory syncytial virus” or “human respiratory syncytial virus”, and “mathematical model” or “dynamic transmission model” or “dynamic model” or “compartment model” or “compartmental model”) and MeSH (“respiratory syncytial virus, human” or “respiratory syncytial viruses”, and “models, theoretical”) search terms. Search terms were applied to all fields, all dates were included, no language restrictions were applied, and only published manuscripts were included.

**Fig. 1:**
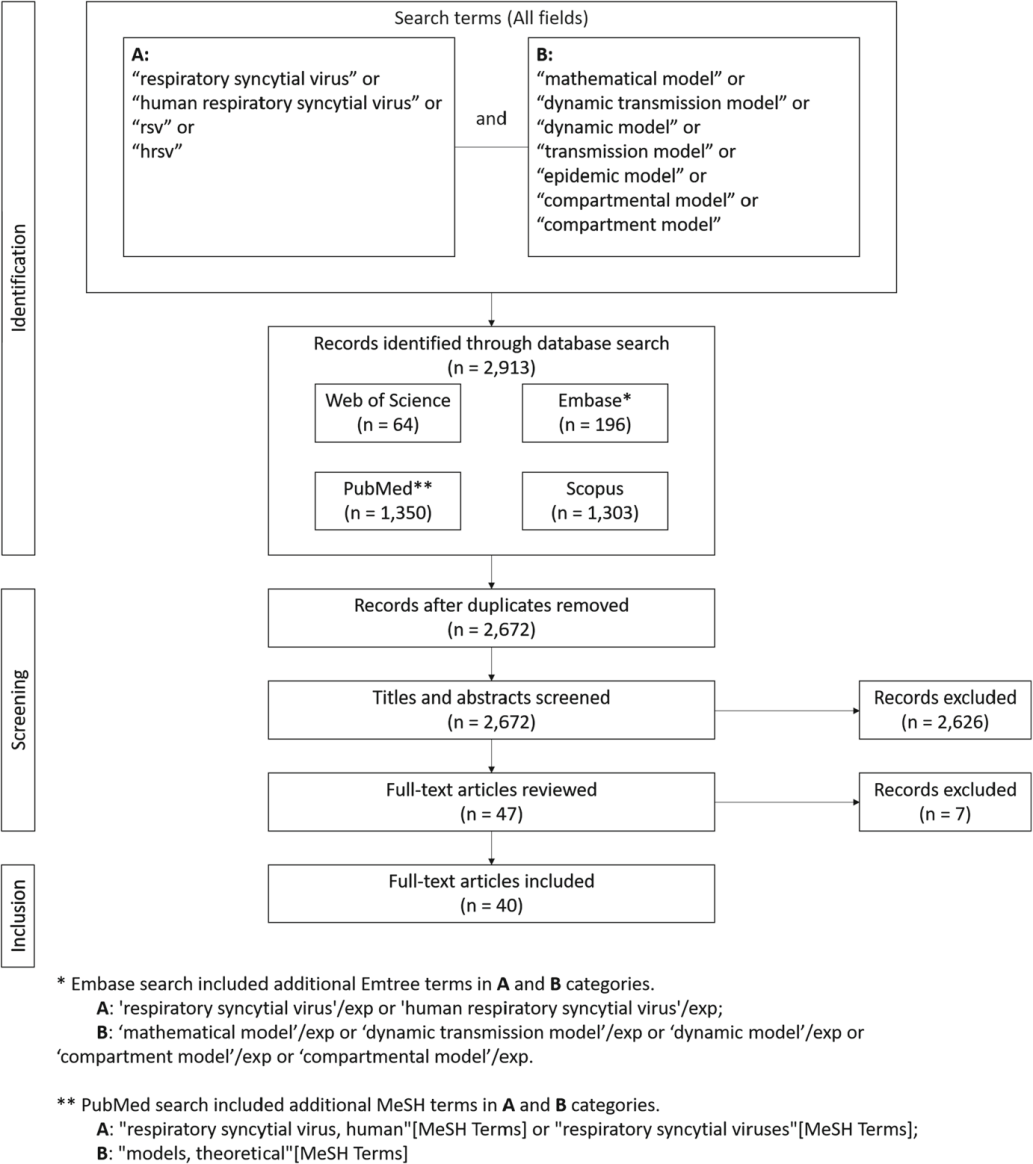
Search strategy for idenitfying RSV DTMs

Duplicates, which were determined by exact match of title, authors, and year of publication, were removed. Subsequently, titles and abstracts were reviewed and a priori inclusion/exclusion criteria were applied. Inclusion criteria are manuscripts that have been published in peer reviewed journals and present a human epidemiologic RSV DTM (e.g., animal and immunologic models are not included). Multi-pathogen DTMs (e.g., a DTM modelling RSV and influenza concurrently) and ensemble models are excluded, as are manuscripts whose primary purpose is other than dynamic transmission modelling of RSV (e.g., a manuscript whose primary focus is the analysis of an abstract DTM in a general context, for which an RSV DTM is given as an example in passing). Full-text articles were retrieved for all manuscripts identified in title and abstract screening procedure. All full-text articles were reviewed in full; inclusion/exclusion criteria were re-applied during review of full-text articles. The sole author of this study performed all search steps in duplicate using Endnote X9 reference management software. The search strategy is summarized in Fig. 1. A summary of data abstracted is given in Table 1. Data abstraction and verification were performed manually and in duplicate by the sole author of this manuscript; no data abstraction software was used.

**Table 1:** Summary of data abstracted

Data abstracted	Description	Summary table(s)
Disease state structure	Record the disease state structure used in the RSV DTM, e.g., *SIR*, *SIRS*, *SEIRS*, etc.	Table 3
Modelling approach	Record the mathematical modelling approach used in the implementation of the RSV DTM, e.g., ordinary differential equation (ODE), stochastic differential equation (SDE), or agent-based model (ABM), etc.	Table 3
Demographic model	Record whether a demographic model is present	Table 3 and Supplemental Table A.2.1
Age strata and ageing rates	Record age strata and the rate at which individuals age from one stratum to the next	Supplemental Table A.2.1
Interventions	Record type, timing, effective coverage, duration, and outcomes for interventions. If multiple scenarios are reported, record representative results, i.e., record results achieved under base-case assumptions	Tables 4, 5 and Table A.3.1
Calibration data	Record location, type, age stratification, time period, frequency, and original references for data used in RSV DTM calibration	Supplemental Table A.4.1
Parameter values	Record value and original references (if available) for common RSV DTM parameters	Supplemental Tables A.5.1–A.5.7
Results	Record major results and findings of RSV DTMs	Supplemental Table A.6.1

### Search results

There were 64, 1303, 196, and 1350 entries retrieved from Web of Science, Scopus, Embase, and PubMed searches, respectively. All searches were performed on December 01, 2020. Following removal of duplicates, titles and abstracts of the 2672 remaining entries were reviewed. Application of inclusion/exclusion criteria resulted in the exclusion of 2626 entries. Full-text manuscripts for the remaining 47 entries were retrieved and reviewed. Application of inclusion/exclusion criteria resulted in the exclusion of seven manuscripts (Capistrán et al. 2009; Guerrero-Flores et al. 2019; Jajarmi et al. 2020; Jódar et al. 2008; Reis et al. 2019; Villanueva-Oller et al. 2013; Zhang et al. 2012). The remaining 40 full-text manuscripts were included in this literature review; two manuscripts were otherwise identified and included (Goldstein et al. 2018; Nugraha and Nuraini 2017).

## RSV DTM structures

### RSV disease state structure

**Fig. 2:**
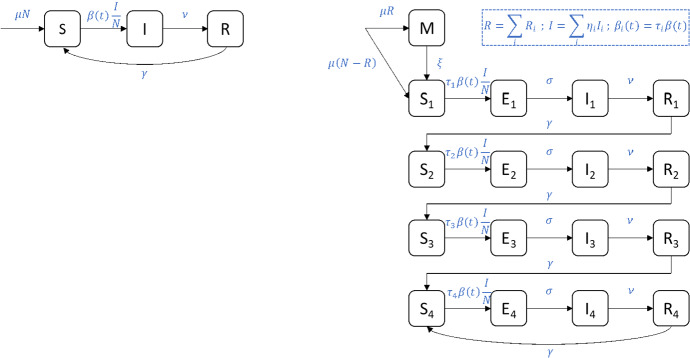
Disease state structure for (left) *SIRS* and (right) *M*-*SEIRS*4 RSV DTMs. Deaths, which occur from all compartments at a rate equal to the birth rate $\mu$, are omitted for clarity. The total population (*N*) is constant. The transmission term $\beta (t)$ is a one year periodic function

The dominant paradigm for disease state structure of RSV DTMs is established in the seminal manuscript of Weber et al. (2001). Specifically, two disease state structures are considered: a simple susceptible-infectious-recovered-susceptible (*SIRS*) disease state structure, and a more complex *M*-*SEIRS*4 model structure (see Fig. 2 and below for definition).

The *SIRS* model partitions individuals into three compartments: susceptible (*S*), infectious (*I*), and recovered (*R*). Infants are born into the susceptible compartment at birth rate $\mu$ and all compartments are subject to natural death at a rate equal to the birth rate, i.e., a constant population is assumed. There are three remaining transitions between compartments: susceptible individuals become infectious through contact with infectious individuals, infectious individuals recover at rate $\nu$ with full temporary immunity to reinfection, and recovered individuals become susceptible at rate $\gamma$ as full temporary immunity wanes. To account for the periodic nature of RSV epidemics, infection is assumed to occur as a result of mass action homogeneous mixing between susceptible and infectious individuals at a periodic time-varying rate proportional to

1 $$\begin{aligned} \beta (t) = b_0 \left( 1+ b_1 \cos (2\pi t-\phi ) \right) \ , \end{aligned}$$

where parameters $b_0$, $b_1$, and $\phi$ represent the average transmission rate, the relative amplitude of seasonal fluctuations in the transmission rate, and the phase shift of the transmission rate, respectively. In other words, new infections occur at the rate $\beta (t)SI/N$, where *N* is the total population.

The *M*-*SEIRS*4 model structure represents a refinement of the *SIRS* structure based on several additional assumptions on the natural history of RSV. First, it assumes that repeated reinfection with RSV results in increasing levels of permanent partial immunity to reinfection. In contrast with full immunity, partial immunity admits reinfection (albeit at a reduced rate). In other words, a susceptible individual with zero, one, two, or three or more previous RSV infections are subdivided into compartments $S_1$, $S_2$, $S_3$, and $S_4$, respectively. Individuals in these compartments become infected at rates proportional to $\tau _1\beta (t)$, $\tau _2 \beta (t)$, $\tau _3 \beta (t)$, and $\tau _4 \beta (t)$, respectively, where $\tau _i$ are the relative susceptibilities of the different susceptible compartments. Second, as with the *SIRS* model, infection is assumed to occur as a result of mass action homogeneous mixing between susceptible and infectious individuals, where the infectiousness of an individual with $i-1$ prior RSV infections is accounted for by multiplication with the relative infectiousness parameter $\eta _i$. In other words, new infections of individuals with $i-1$ prior RSV infections occur at the rate $\beta (t) \tau _i S_i \sum _j \eta _j I_j/N$. Third, a latency period (i.e., compartment $E_i$) is assumed where individuals are infected but not yet infectious, from which infectiousness emerges at rate $\sigma$. Finally, it is assumed that individuals are born either susceptible ($S_1$) or with full temporary immunity to RSV infection due to transfer of natural maternal antibodies (*M*). Individuals born with natural maternal antibodies are said to have natural maternal immunity (NMI) and enter compartment *M* at a rate proportional to the fraction of recovered individuals in the population. Natural maternal immunity wanes at rate $\xi$ and results in individuals becoming susceptible. A summary of parameter and compartment definitions for the *SIR* and *M*-*SEIRS*4 models is provided in Table 2.

**Table 2:** Common disease states and parameters of RSV DTMs

	Description
*Disease compartments*	
*M*	Natural maternal immunity
*S*	Susceptible
*E*	Exposed
*I*	Infectious
*R*	Recovered
*Parameters*	
$\mu$	Birth/death rate
$\xi$	Natural maternal immunity waning rate
$b_0$	Average transmision rate
$b_1$	Relative amplitude of seasonal fluctuations in the transmission rate
$\phi$	Phase shift of the transmission rate
$\tau$	Relative susceptibility to RSV infection
$\eta$	Relative infectiousness while infectious with RSV
$\sigma$	Rate of emergence of infectiousness
$\nu$	Recovery rate
$\gamma$	Immunity waning rate

The *SIRS* and *M*-*SEIRS*4 models described above are antecedent to the majority of identified RSV DTMs and admit the (*M*)-*XXXXn* notation for similar disease state structures. If present, the prefix *M*- is used to indicate that some infants are born with NMI; otherwise, all infants are born without NMI. The body *XXXX* is used to indicate the compartments for the progression of RSV infection. The suffix *n* indicates the number of levels of partial immunity to RSV that are granted due to repeat reinfections.

Whereas disease progression for the RSV DTMs that conform to the (*M*)-*XXXXn* notation follow a simple linear pattern, disease progression in non-standard RSV DTMs are complicated by one or more of the following elements: (a) separate compartments for RSV groups A and B (Kombe et al. 2019; White et al. 2005), (b) multiple types of infectious compartments (Hodgson et al. 2020; Kinyanjui et al. 2020; Kombe et al. 2019; Mahikul et al. 2019; Pan-Ngum et al. 2017; Yamin et al. 2016), (c) waning of partial immunity to reinfection by RSV for susceptible individuals (Kinyanjui et al. 2020; Mahikul et al. 2019; Pan-Ngum et al. 2017; White et al. 2005, 2007), or (d) multiple nested dynamic transmission models (Arenas et al. 2008; White et al. 2007).

A summary of models by their disease structure is provided in Table 3. For completeness, a brief summary of differences in the implementation of NMI between RSV DTMs is provided in Supplementary Materials 1: Appendix A.1.

**Table 3:** Summary of RSV DTM structures

Year	Reference	Modelling approach	Country	Age stratification	Demographic model	Intervention
*SIRSn* *models*						
2001	Weber et al. (2001)	ODE	Finland, The Gambia, Singapore, USA	N/A	N/A	N/A
2009	Arenas et al. (2009)	SDE	Spain	N/A	N/A	N/A
2010	Acedo et al. (2010a)	ODE	Spain	Present	Present	Vaccination at birth
2010	Acedo et al. (2010b)	ODE	Spain	Present	Present	Vaccination of infants
2010	Arenas et al. (2010)	ODE	Spain	N/A	N/A	N/A
2011	Ponciano and Capistrán (2011)	ODE	Finland, The Gambia	N/A	N/A	N/A
2013	Aranda-Lozano et al. (2013)	ODE	Colombia	N/A	N/A	N/A
2014	Corberán-Vallet and Santonja (2014)	S$\varDelta$E	Spain	N/A	N/A	Vaccination of infants
2015	Morris et al. (2015)	ODE	N/A	N/A	N/A	N/A
2017	Nugraha and Nuraini (2017)	ODE	USA	N/A	N/A	Vaccination at birth, Public awareness campaign
2017	Jornet-Sanz et al. (2017)	S$\varDelta$E	Spain	N/A	N/A	N/A
2017	Smith et al. (2017)	ODE	N/A	N/A	N/A	Maternal vaccination, Vaccination (all ages)
2018	Rosa and Torres (2018b)	ODE	USA	N/A	N/A	Other treatment
2018	Rosa and Torres (2018a)	FDE	USA	N/A	N/A	Other treatment
*M*-*SIRSn* *models*						
2015	Kinyanjui et al. (2015)	ODE	Kenya	Present	Present	Maternal vaccination, Vaccination of infants, Vaccination of school-aged children
2015	Poletti et al. (2015)	ABM	Kenya	Present	Present	Vaccination ($\le 10$-month-olds)
2017	Pan-Ngum et al. (2017)	ODE	Kenya	Present	Present	Maternal vaccination, Vaccination of infants
2020	Brand et al. (2020)	ODE	Kenya	Present	Present	Maternal vaccination, Vaccination of households with newborns
2020	Kinyanjui et al. (2020)	ODE	United Kingdom	Present	Present	Vaccination of infants
*SEIRS* *models*						
2016	Paynter (2016)	ODE	Philippines	N/A	N/A	N/A
2018	Rosa and Torres (2018b)	ODE	USA	N/A	N/A	Other treatment
2018	Rosa and Torres (2018a)	FDE	USA	N/A	N/A	Other treatment
*SEIRSn* *models*						
2014	Moore et al. (2014)	ODE	Australia	Present	Present	N/A
2014	Paynter et al. (2014)	ODE	Philippines	N/A	N/A	N/A
2016	Hogan et al. (2016)	ODE	Australia	Present	Present	N/A
2019	Arguedas et al. (2019)	ODE	Mexico	Present	Present	N/A
*M*-*SEIRSn* *models*						
2001	Weber et al. (2001)	ODE	Finland, The Gambia, Singapore, USA	N/A	N/A	N/A
2017	Hogan et al. (2017)	ODE	Australia	Present	Present	Maternal vaccination
2020	Campbell et al. (2020)	ABM	Australia	Present	Present	Maternal vaccination
*SIS* *models*						
2011	Mwambi et al. (2011)	S$\varDelta$E	Kenya	N/A	N/A	N/A
*M*-*SIS*4 *models*						
2015	Pitzer et al. (2015)	ODE	USA	Present	Present	N/A
*SIR* *models*						
2016	Reis and Shaman (2016)	ODE	USA	N/A	N/A	N/A
2018	Goldstein et al. (2018)	$\varDelta$E	USA	Present	Present	Vaccination (various ages)
2018	Reis and Shaman (2018)	ODE	USA	N/A	N/A	N/A
2019	Baker et al. (2019)	S$\varDelta$E	Mexico, USA	N/A	N/A	N/A
2020	Seroussi et al. (2020)	ODE	USA	N/A	N/A	N/A
2020	van Boven et al. (2020)	ODE	The Netherlands	Present	N/A	Maternal vaccination, Vaccination of infants
*SEIR* *models*						
2011	Leecaster et al. (2011)	ODE	USA	Present	Present	N/A
*Other models*						
2005	White et al. (2005)	ODE	Finland, United Kingdom	N/A	N/A	N/A
2007	White et al. (2007)	ODE	Brazil, Finland, The Gambia, Singapore, Spain, United Kingdom, USA	N/A	N/A	N/A
2008	Arenas et al. (2008)	ODE	Brazil, Spain	N/A	N/A	N/A
2016	Yamin et al. (2016)	$\varDelta$E	USA	Present	Present	Vaccination (various ages)
2017	Pan-Ngum et al. (2017)	ODE	Kenya	Present	Present	Maternal vaccination, Vaccination of infants
2019	Kombe et al. (2019)	ABM	Kenya	Present	Present	N/A
2019	Mahikul et al. (2019)	ODE	Thailand	Present	Present	N/A
2020	Hodgson et al. (2020)	ODE	United Kingdom	Present	N/A	Maternal vaccination, Vaccination of infants, Vaccination of children, Vaccination of older adults, Monoclonal antibody immunoprophylaxis
2020	Kinyanjui et al. (2020)	ODE	United Kingdom	Present	Present	Vaccination of infants

*N/A* not applicable, *ODE* ordinary differential equation, $\varDelta \; E$ discrete difference equation, *SDE* stochastic differential equation, S$\varDelta \; E$ stochastic difference equation, *ABM* agent-based model, *FDE* fractional differential equation

### Demographic model structure

Demographic model structure is principally incorporated through stratification of the population by age. With the exception of three agent-based models (Campbell et al. 2020; Kombe et al. 2019; Poletti et al. 2015), and two models restricted to $< 2$-year-olds (Hogan et al. 2016; Paynter 2016), age stratification uses a finer resolution for young children ($< 5$-year-olds) and a coarser resolution for adolescents, adults, and older adults (Acedo et al. 2010a, b; Arguedas et al. 2019; Brand et al. 2020; Goldstein et al. 2018; Hodgson et al. 2020; Hogan et al. 2016, 2017; Kinyanjui et al. 2015, 2020; Kombe et al. 2019; Leecaster et al. 2011; Mahikul et al. 2019; Moore et al. 2014; Pan-Ngum et al. 2017; Paynter et al. 2014; Pitzer et al. 2015; Poletti et al. 2015; van Boven et al. 2020; Yamin et al. 2016). The majority of transitions between age strata are implemented at a rate proportional to the inverse of the width of the age strata of origin (Arguedas et al. 2019; Brand et al. 2020; Hodgson et al. 2020; Hogan et al. 2016, 2017; Kinyanjui et al. 2015, 2020; Leecaster et al. 2011; Moore et al. 2014; Pan-Ngum et al. 2017; Pitzer et al. 2015; van Boven et al. 2020). Alternatively, some RSV DTMs implement more complicated ageing schemes to maintain realistic age structures (Acedo et al. 2010a, b; Kinyanjui et al. 2020; Yamin et al. 2016). A few models provide additional demographic structure through organization of the simulation population by household (Brand et al. 2020; Campbell et al. 2020; Kombe et al. 2019; Mahikul et al. 2019), household and primary school (Poletti et al. 2015), and geography (Seroussi et al. 2020). A more detailed discussion of demographic structure is presented in Supplemental Materials 1: Appendix A.2.

### Interventions

The most common intervention considered is vaccination or monoclonal immunoprophylaxis that induces full temporary immunity to RSV infection (Acedo et al. 2010a, b; Brand et al. 2020; Goldstein et al. 2018; Hodgson et al. 2020; Jornet-Sanz et al. 2017; Kinyanjui et al. 2015; Nugraha and Nuraini 2017; van Boven et al. 2020); however, vaccination inducing partial temporary immunity to RSV infection (Hogan et al. 2017; Kinyanjui et al. 2020; Pan-Ngum et al. 2017; Smith et al. 2017; Yamin et al. 2016), public awareness campaigns (Nugraha and Nuraini 2017), and treatment (Rosa and Torres 2018a, b), are also considered. Interventions are generally assumed to occur uniformly throughout the year, however, exceptions include models that assume vaccination occurs at arbitrary time points throughout the year (Smith et al. 2017), seasonally according to the pattern observed in influenza vaccination (Yamin et al. 2016), at enrollment of primary school (Poletti et al. 2015), and prior to the RSV season (Goldstein et al. 2018; Hodgson et al. 2020). Target populations are typically newborn and infants (Acedo et al. 2010a, b; Hodgson et al. 2020; Hogan et al. 2017; Jornet-Sanz et al. 2017; Kinyanjui et al. 2015, 2020; Nugraha and Nuraini 2017; Pan-Ngum et al. 2017; Poletti et al. 2015; Smith et al. 2017; van Boven et al. 2020), or young children (Hodgson et al. 2020; Poletti et al. 2015), however, vaccination of all age strata are also considered (Goldstein et al. 2018; Hodgson et al. 2020; Yamin et al. 2016). Maternal vaccination is also sometimes considered (Brand et al. 2020; Campbell et al. 2020; Hodgson et al. 2020; Hogan et al. 2017; Pan-Ngum et al. 2017; Poletti et al. 2015; Smith et al. 2017; van Boven et al. 2020), however, the effect of maternal vaccination on the mother is frequently omitted (Hogan et al. 2017; Pan-Ngum et al. 2017; Smith et al. 2017; van Boven et al. 2020). Additional details on model interventions are included in Supplemental Materials 1: Appendix A.3.

### Modelling techniques

Whereas the majority of models are implemented as ordinary differential equation (ODE)-type models (Acedo et al. 2010a; Aranda-Lozano et al. 2013; Arenas et al. 2008, 2010; Arguedas et al. 2019; Brand et al. 2020; Hodgson et al. 2020; Hogan et al. 2016, 2017; Kinyanjui et al. 2015, 2020; Leecaster et al. 2011; Mahikul et al. 2019; Moore et al. 2014; Morris et al. 2015; Nugraha and Nuraini 2017; Pan-Ngum et al. 2017; Paynter 2016; Paynter et al. 2014; Pitzer et al. 2015; Ponciano and Capistrán 2011; Reis and Shaman 2016, 2018; Rosa and Torres 2018b; Seroussi et al. 2020; Smith et al. 2017; van Boven et al. 2020; Weber et al. 2001; White et al. 2005, 2007), there have also been RSV DTMs implemented as stochastic differential equation (SDE) models (Arenas et al. 2009), discrete difference equation ($\varDelta$E) models (Goldstein et al. 2018; Yamin et al. 2016), stochastic difference equation models (S$\varDelta$E) (Baker et al. 2019; Corberán-Vallet and Santonja 2014; Jornet-Sanz et al. 2017; Mwambi et al. 2011), agent-based models (ABMs) (Acedo et al. 2010b; Campbell et al. 2020; Kombe et al. 2019; Poletti et al. 2015), and fractional differential equation (FDE) models (Rosa and Torres 2018a), see Table 3.

ODE and $\varDelta$E models use a deterministic modelling approach that is specified in continuous and discrete time, respectively. These approaches are relatively well understood, can be solved relatively quickly (i.e., with low computational cost), and are easily adapted to many dynamic systems. These models perform best at predicting the average outcome under the assumption of a large well-mixed population.

SDE and S$\varDelta$E models are extensions of ODE and $\varDelta$E models, respectively, that incorporate random effects. For example, two SDE models were developed in order to study interseason variance in RSV epidemics (Arenas et al. 2009). Similarly, S$\varDelta$E models have been developed to study infection dynamics when only small numbers of infectious individuals are present (Corberán-Vallet and Santonja 2014; Jornet-Sanz et al. 2017). Alternatively, whereas other models require the specification of a functional form for the time-varying transmission rate $\beta (t)$, two S$\varDelta$E models have been developed to estimate (a) the transmission rate $\beta (t)$ as a function of time (Mwambi et al. 2011), and (b) both the number of susceptible individuals and the transmission rate $\beta (t)$ as a function of time (Baker et al. 2019).

ABMs are characterized by their specification of rules for the behaviors of individual agents. ABMs admit a granular demographic structure that is generally not considered in standard ODE models, e.g., they are capable of organizing the population into households (Campbell et al. 2020; Kombe et al. 2019), or households and primary schools (Poletti et al. 2015). However, ABMs can be limited by insufficient data for proper model calibration and parameterization, and by the high computational cost of simulations.

Finally, FDE models represent a new non-local modelling approach that introduces a form of “memory” (Du et al. 2013), in which the future evolution of a FDE model simultaneously depends upon its present state and its past states. An initial FDE model has been proposed (Rosa and Torres 2018a), however, it is unclear what advantages, if any, exist that would justify the additional complexity of FDE models over the alternatives proposed above.

## Parameterization and calibration

The RSV DTMs summarized above have been calibrated to diverse data sets collected from more than a dozen countries, i.e., Australia (Campbell et al. 2020; Hogan et al. 2016, 2017; Moore et al. 2014), Brazil (White et al. 2007), Colombia (Aranda-Lozano et al. 2013), Finland (Ponciano and Capistrán 2011; Weber et al. 2001; White et al. 2005, 2007), The Gambia (Ponciano and Capistrán 2011; Weber et al. 2001; White et al. 2007), Kenya (Brand et al. 2020; Kinyanjui et al. 2015; Kombe et al. 2019; Pan-Ngum et al. 2017; Poletti et al. 2015), Mexico (Arguedas et al. 2019; Baker et al. 2019), The Netherlands (van Boven et al. 2020), Philippines (Paynter et al. 2014), Singapore (Weber et al. 2001; White et al. 2007), Spain (Acedo et al. 2010a, b; Arenas et al. 2009, 2010; Corberán-Vallet and Santonja 2014; Jornet-Sanz et al. 2017; White et al. 2007), Thailand (Mahikul et al. 2019), the United Kingdom (Hodgson et al. 2020; Kinyanjui et al. 2020; White et al. 2005, 2007), and the United States (Baker et al. 2019; Goldstein et al. 2018; Leecaster et al. 2011; Nugraha and Nuraini 2017; Pitzer et al. 2015; Reis and Shaman 2016, 2018; Rosa and Torres 2018a, b; Seroussi et al. 2020; Weber et al. 2001; White et al. 2007; Yamin et al. 2016). These data mostly consist of RSV detected in inpatient settings only, i.e., hospitalizations, or in inpatient and outpatient settings. One model uses Google searches for the term “RSV” as a proxy for the number of RSV infections (Seroussi et al. 2020). Data have been gathered for infants ($< 1$-year-olds) (Acedo et al. 2010a, b; Campbell et al. 2020; Corberán-Vallet and Santonja 2014; Jornet-Sanz et al. 2017; White et al. 2005), toddlers ($< 2$-year-olds) (Hogan et al. 2016, 2017; Moore et al. 2014; Paynter et al. 2014; Ponciano and Capistrán 2011; Weber et al. 2001; White et al. 2007), young children ($< 5$-year-olds) (Aranda-Lozano et al. 2013; Arenas et al. 2009, 2010; Hodgson et al. 2020; Kinyanjui et al. 2015, 2020; Pan-Ngum et al. 2017; Poletti et al. 2015; White et al. 2007), children (Leecaster et al. 2011; Hodgson et al. 2020; Nugraha and Nuraini 2017; Ponciano and Capistrán 2011; Weber et al. 2001; White et al. 2005, 2007), and the entire population (Arguedas et al. 2019; Baker et al. 2019; Brand et al. 2020; Goldstein et al. 2018; Hodgson et al. 2020; Kombe et al. 2019; Mahikul et al. 2019; Pitzer et al. 2015; Reis and Shaman 2016, 2018; Seroussi et al. 2020; van Boven et al. 2020; Weber et al. 2001; White et al. 2007; Yamin et al. 2016). Frequency of measurements are daily (Leecaster et al. 2011), biweekly (Kombe et al. 2019), weekly (Acedo et al. 2010a, b; Aranda-Lozano et al. 2013; Arguedas et al. 2019; Brand et al. 2020; Corberán-Vallet and Santonja 2014; Hodgson et al. 2020; Hogan et al. 2016; Jornet-Sanz et al. 2017; Kinyanjui et al. 2020; Moore et al. 2014; Pitzer et al. 2015; Poletti et al. 2015; Ponciano and Capistrán 2011; Reis and Shaman 2016, 2018; Seroussi et al. 2020; van Boven et al. 2020; Weber et al. 2001; White et al. 2005, 2007; Yamin et al. 2016), monthly (Arenas et al. 2009, 2010; Hogan et al. 2017; Kinyanjui et al. 2015; Moore et al. 2014; Mahikul et al. 2019; Nugraha and Nuraini 2017; Pan-Ngum et al. 2017; Ponciano and Capistrán 2011; Rosa and Torres 2018a, b; Weber et al. 2001; White et al. 2007), or annually (Goldstein et al. 2018; White et al. 2005). For additional details, see Supplemental Materials 1: Appendix A.4.

This review has compiled values for comparison of common model parameters determined through calibration or literature search. Comparison of parameter values has value in not only populating future RSV DTMs, but also in identifying uncertainty in aspects of the natural history of RSV that may require further research to resolve. Results for four common parameters are summarized: NMI waning rate ($\xi$), relative susceptibility to RSV infection ($\tau$), recover rate ($\nu$), and immunity waning rate ($\gamma$). A comprehensive summary of common parameter values is provided in Supplementary Materials 1: Appendix A.5.

### Natural immunity waning rate ($\xi$)

Seven models estimate NMI waning rate from literature values. For the five most recent models the NMI waning rate lies within the range 2.7–4.1 per year, equivalent to a duration of 90–134 days (Campbell et al. 2020; Hodgson et al. 2020; Pitzer et al. 2015; Poletti et al. 2015; Yamin et al. 2016); for the remaining two models the NMI waning rate is 13.0 per year, equivalent to a duration of 28 days (Arenas et al. 2009; Weber et al. 2001). In contrast, the calibration of six models produces estimates of the NMI waning rate in the range 5.2–49.6 per year, equivalent to a duration of 7–70 days (Brand et al. 2020; Kinyanjui et al. 2015, 2020; Pan-Ngum et al. 2017). Comparison of these values indicate some uncertainty exists in the NMI waning rate which may require additional research to resolve.

### Relative susceptibility to RSV infection ($\tau$)

Five models estimate relative susceptibility of individuals with at least one previous RSV infection ($\tau _1$) to be in the range 0.45–0.77, when measured with respect to the reference susceptibility of RSV naïve individuals ($\tau _0 = 1$) (Brand et al. 2020; Kinyanjui et al. 2020; Mahikul et al. 2019; Morris et al. 2015; Paynter et al. 2014). In contrast, calibration of two models produces estimates in the range 0.68–0.88 (Poletti et al. 2015; White et al. 2007). These values are largely consistent and give insight into the approximate range for relative susceptibility of individuals previously infected with RSV.

### Recovery rate ($\nu$)

Using literature values, twenty three papers estimate the recovery rate to be in the range 33.2–46.8 per year, equivalent to a duration of 8–11 days (Acedo et al. 2010a, b; Aranda-Lozano et al. 2013; Arenas et al. 2008, 2009, 2010; Campbell et al. 2020; Corberán-Vallet and Santonja 2014; Goldstein et al. 2018; Hogan et al. 2016, 2017; Jornet-Sanz et al. 2017; Leecaster et al. 2011; Moore et al. 2014; Nugraha and Nuraini 2017; Poletti et al. 2015; Ponciano and Capistrán 2011; Rosa and Torres 2018a, b; Smith et al. 2017; Weber et al. 2001; White et al. 2005, 2007). In contrast, calibration of two models produces estimates in the range 57.0–70.2 per year, equivalent to a duration of 5–6 days (Reis and Shaman 2016, 2018). As with the NMI waning rate, the discrepancy between literature and calibration estimates may indicate some uncertainty in the recovery rate; however, it is noted that the models that estimate recovery rate through calibration employ an *SIR* model structure to model each season separately, i.e., they depart from the standard (*M*)-*XXXXn* disease structure typically employed in RSV DTMs.

### Immunity waning rate ($\gamma$)

Nineteen papers use literature values to estimate a range for the immunity waning rate: 1.8–2.0 per year, equivalent to a duration of 183–203 days (Acedo et al. 2010a, b; Aranda-Lozano et al. 2013; Arenas et al. 2009, 2010; Brand et al. 2020; Corberán-Vallet and Santonja 2014; Jornet-Sanz et al. 2017; Kinyanjui et al. 2015, 2020; Morris et al. 2015; Nugraha and Nuraini 2017; Pan-Ngum et al. 2017; Ponciano and Capistrán 2011; Rosa and Torres 2018a, b; Smith et al. 2017; Weber et al. 2001; Yamin et al. 2016). One model uses literature to estimate a rate of 5.8 per year, equivalent to a duration of 63 days (Paynter et al. 2014). One model uses literature to estimate a rate of 1.0 per year, equivalent to a duration of 359 days (Hodgson et al. 2020). In contrast, calibration of three models produces estimates of the immunity waning rate in the range 1.6–2.1 per year, equivalent to 171–230 days (Hogan et al. 2016; Moore et al. 2014; Poletti et al. 2015). These values are largely consistent and give insight into an approximate range for immunity waning rate.

## Modelling results

In this section we summarize some important modelling results of the RSV DTMs reviewed above. For additional details see Supplementary Materials 1: Appendix A.6.

### General modelling results

The *SIRS* and *M*-*SEIRS*4 RSV DTMs introduced above (Weber et al. 2001) (see Fig. 2) establish a disease state structure that informs, directly or indirectly, the disease state structure of most subsequent RSV DTMs. A sensitivity analysis performed on the *SIRS* ODE model by varying initial conditions, birth rate ($\mu$), and average transmission parameter ($b_0$) finds that the model is least sensitive to uncertainty in initial conditions and most sensitive to uncertainty in average transmission parameter (Arenas et al. 2010). Consistent results are reported for an *SIRS* SDE models (Arenas et al. 2009).

Hogan and colleagues performed an analysis of an age-stratified *SEIRS* ODE model that provides some insight into the behavior of models implementing the (*M*)-*XXXXn* disease state structure (Hogan et al. 2016). The simple *SEIRS* model was able to reproduce the diverse periodic behaviors observed in RSV epidemics: an annual pattern of repeating peaks, a biennial pattern of repeating high followed by low peaks where peaks occur at the same time each year, and a biennial pattern of high followed by low peaks where high peaks occur earlier in the year than low peaks. Roughly speaking, annual peaks result when the duration of immunity ($1/\gamma$) is short, the former biennial pattern results when the average transmission coefficient ($b_1$) is large, and the latter biennial pattern for intermediate values of the birth rate ($\mu$).

Additional insight into the (*M*)-*XXXXn* disease structure is provided by comparing a system of eight standard nested models (including, e.g., *SIS*, *SIR*, and *SIRS* model structures, among others) on their ability to reproduce RSV epidemic data (White et al. 2007). The most parsimonious model with the best fit was a model with partial permanent immunity following an initial infection with RSV. These results are consistent with the majority of models that implement the (*M*)-*XXXXn* disease structure, a structure with increasing levels of partial permanent immunity resulting from repeated RSV infections. Additional evidence supporting the inclusion of partial permanent immunity following initial infection with RSV is provided by Morris and colleagues (Morris et al. 2015), who conclude that the *SIRS*2 model is better able to capture sensitivity of RSV epidemics to birth rate than the *SIRS* model.

### Results for RSV interventions

Direct comparison of modelling results is complicated by several factors. First, different parameter ranges are considered for vaccine effective coverage (the product of vaccine coverage and effectiveness), and duration of protection. Second, models differ by the mechanism of protection (e.g., full temporary immunity versus partial temporary immunity). Third, outcomes are measured with respect to different populations. Comparison of modelling results are, therefore, qualitative in nature.

Seven models report reduction in hospitalizations or infections due to maternal vaccination (Brand et al. 2020; Campbell et al. 2020; Hodgson et al. 2020; Hogan et al. 2017; Pan-Ngum et al. 2017; Poletti et al. 2015; van Boven et al. 2020). These models exhibit four different mechanisms of protection: full temporary immunity provided to both mother and infant (Brand et al. 2020; Hodgson et al. 2020; Poletti et al. 2015), full temporary immunity provided to mother and partial temporary immunity provided to infant (Campbell et al. 2020), full temporary immunity provided to infant only (van Boven et al. 2020), and partial temporary immunity provided to infant only (Hogan et al. 2017; Pan-Ngum et al. 2017), see Table 4 for representative results. For effective coverage of 35–60$\%$ and duration of protection of 3–6 months, the reduction in hospitalizations of infants ($< 1$-year-olds) is approximately 6–20$\%$ and the reduction in infections of infants ($< 1$-year-olds) is approximately 17–26$\%$.

**Table 4:** Reduction in hospitalization or infection due to maternal vaccination

Year	Reference	Effective coverage (%)	Duration (months)	Reference population (age in months)	Percent reduction (%)
*Newborns granted PTI*					
2017	Hogan et al. (2017)	40	6	0–2	6–37$^{\mathrm{a}}$
				3–5	30–46$^{\mathrm{a}}$
				6–11	0$^{\mathrm{a}}$
			3	0–2	25$^{\mathrm{a}}$
				3–5	0$^{\mathrm{a}}$
				6–11	0$^{\mathrm{a}}$
2017	Pan-Ngum et al. (2017)	35	3	$<12$	7–15$^{\mathrm{a}}$
*Newborns granted FTI*					
2020	van Boven et al. (2020)	50	6	$<12$	26$^{\mathrm{b}}$
*Newborns granted PTI; mothers granted FTI*					
2020	Campbell et al. (2020)	N/A$^{\mathrm{c}}$	3	$<3$	17$^{\mathrm{b}}$
				3–6	5$^{\mathrm{b}}$
*Newborns and mothers granted FTI*					
2015	Poletti et al. (2015)	60	6	$<12$	17$^{\mathrm{b}}$
2020	Brand et al. (2020)	50	3	$<60$	19$^{\mathrm{a}}$
2020	Hodgson et al. (2020)	32	4	All ages	9$^{\mathrm{a}}$

*PTI* partial temporary immunity, *FTI* full temporary immunity

$^{\mathrm{a}}$Percent reduction in hospitalizations

$^{\mathrm{b}}$Percent reduction in infections

$^{\mathrm{c}}$Coverage was 70%; effectiveness was not specified

Seven models report reduction in hospitalizations or infections due to infant vaccination or monoclonal immunoprophylaxis (Hodgson et al. 2020; Jornet-Sanz et al. 2017; Kinyanjui et al. 2015, 2020; Pan-Ngum et al. 2017; Poletti et al. 2015; van Boven et al. 2020). Analogous to maternal vaccination, these models exhibit two different mechanisms of protection: partial temporary immunity (Kinyanjui et al. 2020; Pan-Ngum et al. 2017), and full temporary immunity (Hodgson et al. 2020; Kinyanjui et al. 2015; Jornet-Sanz et al. 2017; van Boven et al. 2020), see Table 5 for representative results. For effective coverage of 80–90$\%$ and duration of protection of 6–12 months, the reduction of hospitalizations of infants ($< 1$-year-olds) is approximately 50–90$\%$ and the reduction in infections of infants ($< 1$-year-olds) is approximately 30–35$\%$.

**Table 5:** Reduction in hospitalization due to infant vaccination

Year	Reference	Effective coverage (%)	Duration (months)	Reference population (age in months)	Percent reduction (%)
*Infants granted PTI*					
2017	Pan-Ngum et al. (2017)	90	12	$<12$	58–89$^{\mathrm{a}}$
2020	Kinyanjui et al. (2020)	90	12	$<12$	55–56$^{\mathrm{a}}$
*Infants granted FTI*					
2015	Kinyanjui et al. (2015)	80	6	$<6$	51–88$^{\mathrm{a}}$
2015	Poletti et al. (2015)	80	6	$<12$	$35^{\mathrm{b}}$
2017	Jornet-Sanz et al. (2017)	80	6	$<24$	$81^{\mathrm{a}}$
2020	Hodgson et al. (2020)	75	12	All ages	$7^{\mathrm{a}}$
		63$^{\mathrm{c}}$	8$^{\mathrm{c}}$	All ages$^{\mathrm{c}}$	$8^{\mathrm{a,c}}$
2020	van Boven et al. (2020)	50	55	$<12$	$30^{\mathrm{b}}$

*PTI* partial temporary immunity, *FTI* full temporary immunity

$^{\mathrm{a}}$Percent reduction in hospitalizations

$^{\mathrm{b}}$Percent reduction in infections

$^{\mathrm{c}}$Long-acting immunoprophylaxis administered to all infants at birth (if born in-season) or at the beginning of the season (if born out-of-season)

A hybrid approach is studied by Brand et al. (2020), in which maternal vaccination is combined with vaccination of the entire household at birth. Maternal vaccination is assumed to provide newborns with an additional 75 days of protection (for a total of 96 days of protection), vaccination of household members is assumed to provide six months of protection, and protection for both forms of vaccination is assumed to take the form of full temporary immunity. Under these assumptions, an effective coverage of 75% of birth households results in a 50% reduction in RSV hospitalizations of under 5-year-olds.

Three models compare vaccination of multiple age groups (Goldstein et al. 2018; Hodgson et al. 2020; Yamin et al. 2016). These results of these three studies are consistent, i.e., it is found that vaccination of under 5-year-olds is the most efficient strategy for averting RSV infection (Hodgson et al. 2020; Yamin et al. 2016), and vaccination of 3–6-year-olds at the beginning of the RSV season results in the greatest reduction in the initial effective reproduction number (Goldstein et al. 2018).

Finally, two models provide a cost-effectiveness analysis for a hypothetical vaccine for infants in Valencia, Spain (Acedo et al. 2010a, b). These models include hospitalization cost, vaccination cost, and parent/caregiver loss of productivity, and find that cost savings are possible when average parent/caregiver loss of productivity exceeds three days per infant infected with RSV. One model provides a cost-effectiveness analysis for palivizumab and three hypothetical products: a maternal vaccine, a vaccine, and a long-acting monoclonal immunoprophylaxis (Hodgson et al. 2020). This model includes costs for administering the vaccine or immunoprophylaxis, hospitalization, and general practice visits, and calculates the maximum cost-effective purchase price for various comparators, see Supplemental Materials 1: Appendix A.6 for additional details.

### Seasonal drivers

Most models presented in this review assume that there exists some periodic forcing of RSV epidemic dynamics, e.g., see Eq. 1. Three models explore potential drivers of this seasonal forcing in detail (Baker et al. 2019; Paynter et al. 2014; Pitzer et al. 2015). In the Philippines the peak in RSV transmission is found to precede the peak in RSV detections by 49–67 days, and nutritional status and rainfall are identified as two potential drivers of RSV epidemic dynamics (Paynter et al. 2014). In the United States correlation is observed between estimated model parameters and climatic variables of temperature, vapor pressure, precipitation, and potential evapotranspiration (Pitzer et al. 2015). For example, the relative amplitude of seasonal fluctuations in the transmission rate ($b_1$) and the phase shift of the transmission rate ($\phi$) were found to be negatively correlated with mean precipitation and mean vapor pressure, and positively correlated with the amplitude and timing of potential evapotranspiration. Similarly, a more recent modelling paper covering both the United States and Mexico finds an inverse relationship between humidity and log transmission and a positive linear relationship between rainfall and transmission rate (Baker et al. 2019). Additionally, one paper estimates the seasonal transmission rate $\beta (t)$ as a function of time for an RSV epidemic in Kilifi, Kenya, and finds two peaks in transmission (May, and January/February) (Mwambi et al. 2011).

### Forecasting RSV epidemics

Four models were developed with application to forecasting RSV epidemic dynamics (Leecaster et al. 2011; Reis and Shaman 2016, 2018; Seroussi et al. 2020). First, the average transmission coefficient ($b_0$) and epidemic start time estimated from consecutive seasons are found to covary, and are potentially predictive of epidemic size (Leecaster et al. 2011). Second, an *SIR* model calibrated to data in real time is developed as a forecasting model that, four weeks prior to the peak in RSV detections, predicts the magnitude of the peak in RSV detections within 25% approximately 70% of the time (Reis and Shaman 2016, 2018). Finally, a multicompartment *SIR* model for the United States is capable of predicting infection rates and timing of infection peaks with high accuracy in each state for the current season using the first seven weeks of RSV data and parameters estimated from the previous year’s data (Seroussi et al. 2020).

## Research gaps and future steps

The diversity of RSV DTMs and their applications admits numerous opportunities for improvement in our understanding of RSV epidemic dynamics. Below, four areas with significant potential for future work are discussed: alignment of RSV DTMs with immunoprophylactic profiles, understanding sensitivity of results to model structure, evaluation of cost-effectiveness through health economic analysis, and investigating seasonal drivers of RSV epidemics.

### Alignment with immunoprophylactic profiles

Only one immunoprophylactic product, i.e., palivizumab, is currently available for the prevention or treatment of RSV disease. Palivizumab is extremely expensive and is only cost-effective in high-risk communities. Because so few individuals are eligible for palivizumab, static models are typically employed in the evaluation of palivizumab on RSV disease. As such, in the context of vaccines or immunoprophylactic interventions for RSV approved and recommended for widespread use, the RSV DTMs identified above are necessarily limited to implementing hypothetical products. As the profiles of the products under development become more well defined, further alignment with RSV DTMs will be possible. Further stratification of the model to include additional sub-populations of interest may be necessary. For example, none of the current RSV DTMs include compartments for high-risk infants, i.e., very premature infants, infants with CHD, or infants with CLD. In particular, stratification by gestational age may be important when evaluating maternal vaccination strategies, since transfer of maternal antibodies for preterm infants is expected to be incomplete (Rainisch et al. 2020). Analogously, evaluations of interventions targeted at older children and adults should also consider inclusion of high-risk subpopulations, i.e., older adults, institutionalized adults, and immunocompromised adults.

### Health economic analysis

As the development of RSV immunoprophylactic products intended for widespread use continues to advance, health economic analyses will become increasingly important tools for informing public health decision making. Whereas health economic analyses that employ static modelling approaches will continue to play an important role, given the highly contagious nature of RSV, we anticipate increasing demand for analyses that are better suited to address indirect effects or herd immunity effects. In other words, we anticipate increasing demand for health economic analyses based on dynamic modelling approaches, i.e., DTMs. Studies that describe a cost-effectiveness analysis based on an RSV DTMs, a subset of all manuscripts that include an RSV DTM, are identified in only three manuscripts (Acedo et al. 2010a, b; Hodgson et al. 2020). There is significant potential for additional RSV DTMs that evaluate costs related to RSV infection in multiple countries and settings. Additionally, there is potential for future work comparing and contrasting health economic analyses that employ static modelling approaches to those that employ RSV DTMs.

### Sensitivity of results to model structure and parameterization

Whereas inclusion of sensitivity analysis (of model results with respect to model parameterization) has become more common in recent RSV DTM studies, there is little consistency between studies with respect to either (a) the parameters included in the sensitivity analyses or (b) the model outcomes used to measure model sensitivity (Campbell et al. 2020; Hodgson et al. 2020; Hogan et al. 2016, 2017; Kinyanjui et al. 2015, 2020; Kombe et al. 2019; Morris et al. 2015; Pan-Ngum et al. 2017; Poletti et al. 2015; Reis and Shaman 2016, 2018; Rosa and Torres 2018a, b; van Boven et al. 2020; Yamin et al. 2016). For example, whereas some RSV DTM studies include a broad selection of parameters in their sensitivity analyses (Campbell et al. 2020; Hogan et al. 2016, 2017; Kinyanjui et al. 2015; Morris et al. 2015; Rosa and Torres 2018a), others only include parameters related to RSV interventions (Hodgson et al. 2020; Kinyanjui et al. 2020; Pan-Ngum et al. 2017; Yamin et al. 2016) or idiosyncratic model assumptions (Kombe et al. 2019; Poletti et al. 2015). Sensitivity analyses for very complex and highly granular RSV DTMs (including, but not limited to ABMs) are further complicated by the large number of parameters needed for model parameterization. In many cases, parameter values may not be available from the literature at the desired granularity, necessitating additional modelling assumptions. Finally, intermodel comparisons are limited by the diversity in model settings (e.g., countries and time periods under consideration), the range and distribution of included parameters, and the modelling outputs reported (e.g., RSV infections versus hospitalizations).

Limitations of sensitivity analyses of model results with respect to model structure, which are generally evaluated through intermodel comparisons, are analogous to the limitations of sensitivity analyses of model results with respect to model parameterization. Indeed, only six studies investigated the sensitivity of model outputs to model structure by comparing the performance of two or more RSV DTMs (Arenas et al. 2008; Kinyanjui et al. 2020; Pan-Ngum et al. 2017; Rosa and Torres 2018a, b; White et al. 2007). Of these, two studies compared the performance of very closely related RSV DTMs (Rosa and Torres 2018a, b) (i.e., SIRS versus SEIRS disease state structures), two studies considered nested ODE models (Arenas et al. 2008; White et al. 2007) (see Sect. 5.1), and two studies compared model structures where RSV infection resulted in either permament or temporary partial immunity to reinfection (Kinyanjui et al. 2020; Pan-Ngum et al. 2017). As models are developed with common settings, and as they are aligned to common immunoprophylactic product profiles, comparison between model predictions may become a practical strategy to validate models and to achieve insights into the sensitivity of results to model structure and parameterization.

### Age effects and seasonal drivers of RSV

Although many RSV DTMs employ age stratification (see Sect. 3.2), only one RSV DTM has been developed to replicate the age-specific dynamics observed in RSV epidemics (Goldstein et al. 2018). Specifically, the mechnism by which RSV infections in children aged 3-6 years tends to lead infections in other age groups (and especially in those aged $\ge 10$-years-old) has not been studied in detail. The mechanisms by which potential seasonal drivers affect epidemic dynamics have also not been described in detail. In other words, seasonality in RSV DTMs is typically incorporated through an exogenous forcing term, e.g., see Eq. 1, that has an arbitrary functional form. Additional research into the sensitivity of RSV DTM results to the functional form of the seasonal forcing term may lead to improvements in how seasonality is included in RSV DTMs. A better understanding of how to endogenize seasonal drivers of RSV epidemics into RSV DTMs, and how to replicate age effects, may allow for more accurate models, better predictions of changing patterns in RSV epidemics (e.g., due to climate change), and identification of more efficient intervention strategies. Together with more granular surveillance data (e.g., age and location stratified surveillance data), these advances may also significantly enhance RSV epidemic forecasting, and hence, immunoprophylactic intervention strategy and timing.

## Discussion

### Stengths and limitations

This literature review, the first literature review of RSV DTMs, provides a comprehensive summary of RSV DTMs. Broad search terms were used and over 2600 titles and abstracts were reviewed in order to identify 38 full-text manuscripts for inclusion (two additional manuscripts were otherwise identified). The manuscripts included in this review represent a diversity of RSV DTMs and admits a broad overview of RSV DTMs provided along multiple dimensions (e.g., disease state structure, underlying demographic model structure, interventions included, calibration method and data, and modelling techniques applied), and perspectives (e.g., analytical/theoretical, epidemiologic, health economic). Furthermore, the Supplementary Materials that accompany this review are a potentially valuable resource. For example, the Supplementary Materials include (but are not limited to) a detailed description of all data sets used in calibration of RSV DTMs, a detailed description of common parameter values used in parameterization of RSV DTMs, and a detailed description of interventions included in RSV DTMs; where applicable, the original references to these additional data have also been provided.

This review is subject to several limitations. First, because risk of bias and quality for RSV DTMs (and DTMs in general) is context dependent, we do not provide an assessment of the risk of bias or quality of the included RSV DTMs. Indeed, the risk of bias or quality in an RSV DTM depends not only on model structure, input parameters, calibration data, modelling technique, et cetera, but also on the modelling objectives. For example, a simple *SIR* model may be appropriate to the forecasting of RSV epidemics, but it is completely incapable of assessing the impact of maternal vaccination on infant hospitalizations. The lack of a critical appraisal of included studies is not unusual for literature reviews (Munn et al. 2018), and a full risk of bias and quality assessment is left as future work, e.g., as part of a future systematic review with a specific research question. Second, this review remains subject to evidence selection bias. Specifically, although this review conducted a very broad search in multiple databases, the choice to include only RSV DTMs presented in published manuscripts admits the risk of publication bias. Finally, this review was entirely completed by a single author. Although all steps in this review were completed in duplicate, we may still expect a higher error rate in screening manuscripts and data abstraction than if this review were conducted by multiple authors working independently and aggregating their results.

### Conclusions

The numerous vaccines and immunoprophylactic interventions currently under development for prevention of RSV infection have the potential to significantly reduce the burden of RSV in infants in the near future. Mathematical modelling provides a means to better understand the natural history of RSV, to forecast severity of RSV epidemics mid-season, to predict long-term changes in patterns of RSV epidemics, and to evaluate the effectiveness of proposed vaccine and immunoprophylactic interventions. This review has provided an overview of existing RSV DTMs that includes disease state structures, demographic model structure, intervention strategies, and modelling techniques. In both the main text and the Supplementary Materials, a list of RSV epidemic data sources and values of common parameters determined through literature and calibration has been compiled. This work provides a strong foundation for future modelling of RSV epidemics and interventions. Research gaps and areas for future potential work have also been identified. In particular, it is anticipated that RSV DTMs, combined with economic cost-effectiveness evaluations, will play a significant role in shaping decision making in the development and implementation of vaccination and immunoprophylaxis programs.

## Supplementary Information

Below is the link to the electronic supplementary material.

Supplementary file 1 (pdf 838 KB)
