# Supplementary material for: Use of mathematical modelling to assess respiratory syncytial virus epidemiology and interventions: a literature review
Source: J Math Biol. 2022 Feb 26;84(4):26. doi: 10.1007/s00285-021-01706-y (PMC8882104; doi:10.1007/s00285-021-01706-y)
Supplement: Supplementary file 1 — (pdf 838 KB) [file 285_2021_1706_MOESM1_ESM.pdf]

Use of mathematical modelling to assess respiratory syncytial virus epidemiology and interventions: A literature review

Supplementary Materials 1: Appendices

John C. Lang, Associate Principal Scientist, Merck & Co., Inc.

Contents

Appendix A.1: **Protection from and coverage of natural maternal immunity** 2

Appendix A.2: **Demographic model structure** 4

Appendix A.3: **Interventions** 5

Appendix A.4: **Calibration data** 9

Appendix A.5: **Common parameter values** 13

Appendix A.6: **Modelling results** 24

Figures

Supplemental Figure A.1.1: Decision tree for RSV infection of infants. 2

Supplemental Figure A.1.2: Parameter estimates m, q, $p1$, and $p2$ as a function of duration of NMI ($\xi - 1$). .. 3

Tables

Supplemental Table A.1.1: RSV incidence in children less than two years old.[13] 2

Supplemental Table A.2.1: Age stratification in RSV DTMs. 4

Supplemental Table A.3.1: Interventions implemented in RSV DTMs. 5

Supplemental Table A.4.1: RSV epidemic data used in calibration of RSV DTMs. 9

Supplemental Table A.5.1: Parameterization of the natural maternal immunity waning rate (ξ) in RSV DTMs. 13

Supplemental Table A.5.2: Parameterization of relative susceptibility to RSV infection (τ) in RSV DTMs. ... 14

Supplemental Table A.5.3: Parameterization of relative infectiousness to RSV infection (η) in RSV DTMs. .. 16

Supplemental Table A.5.4: Parameterization of rate for emergence of infectiousness (σ) in RSV DTMs. 18

Supplemental Table A.5.5: Parameterization of the recovery rate (ν) in RSV DTMs. 19

Supplemental Table A.5.6: Parameterization of the immunity waning rate (γ) in RSV DTMs. 22

Supplemental Table A.5.7: Parameterization of the social mixing matrix (C). 24

Supplemental Table A.6.1: Summary of results of RSV DTMs. 24

Appendix A.1: Protection from and coverage of natural maternal immunity

The most common assumptions for implementation of natural maternal immunity (NMI) are that the entire birth cohort receives NMI,[1-9] and infants with NMI receive full temporary immunity from infection with RSV.[1-5,7-10] Less commonly, some models have assumed only partial coverage of the birth cohort with NMI,[9-12] and that infants with NMI are only granted partial temporary protection.[6,11,12] Although the dominant assumptions of full coverage and full temporary protection of NMI are not explicitly justified in the modelling literature, they are roughly consistent with RSV incidence data,[13] see below.

In the remainder of this section we demonstrate that the assumptions of (a) full coverage of the birth cohort with NMI, and (b) full temporary protection from RSV infection for infants with NMI, are consistent with RSV incidence data reported by Glezen and colleagues (see Supplemental Table A.1.1).[13] We assume the following:

1. Infants are born with NMI with probability m.
2. The annualized probability that an RSV naïve infant (< 1-year-olds) or 1-year-olds without NMI becomes infected with RSV is p_1.
3. The annualized probability that an RSV naïve infant with NMI becomes infected with RSV is q.
4. The average duration of NMI (ξ^{-1}) is less than one year, i.e., $\xi^{-1} \in [0, 1]$.

Supplemental Table A.1.1: RSV incidence in children less than two years old.[13]

Symbol	Description	Value
n_0	Number of infants	125
k_0	Number of infants infected with RSV in their first season	85
n_1	Number of RSV naïve 1-year-olds	34
k_1	Number of RSV naïve 1-year-olds infected with RSV in their second season	33
n_2	Number of 1-year-olds previously infected with RSV	58
k_2	Number of 1-year-olds re-infected with RSV in their second season	44

From these assumptions we construct the decision tree for the first year of life, see Figure A.1.1. Specifically, infants are born with NMI with probability m and are born without NMI with probability $1 - m$. Infants born without NMI are infected with RSV in their first year of life with probability p_1. Infants born with NMI spend the first ξ^{-1} of their first year of life with NMI; during this period infants are infected with RSV with probability $1 - (1 - q)^{\xi^{-1}}$. Infants born with NMI that are not infected with RSV during the first ξ^{-1} years of their life become RSV naïve for the remainder of their first year of life the probability that they are infected with RSV is $1 - (1 - p_1)^{1-\xi^{-1}}$.

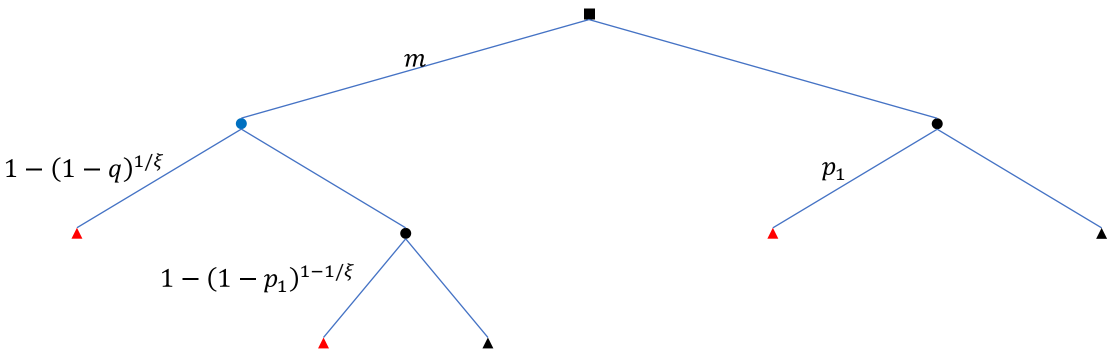

Supplemental Figure A.1.1: Decision tree for RSV infection of infants. *(Black square) root node. (Blue circle) Infant born with NMI. (Black circles) Infants without NMI. (Red triangles) Infants infected with RSV in their first year of life. (Black triangles) Infants that remain RSV naïve after their first year of life.*

It follows from the decision tree in Figure A.1.1 that the probability of becoming infected with RSV in the first year of life is

$$p_0 = (1-m) * p_1 + m * \left(1-(1-q)^{\xi^{-1}}\right) + m * (1-q)^{\xi^{-1}} * \left(1-(1-p_1)^{1-\xi^{-1}}\right).$$

In the second year of life RSV naïve toddlers are infected with probability p_1 and toddlers with previous RSV infection are infected with probability p_2. Given the data in Supplemental Table A.1.1, this allows us to form the log likelihood function

$$ll(m, q, p_1, p_2) = constant + \sum_{i=0}^{2} k_i * \log(p_i) + (n_i - k_i) * \log(1-p_i).$$

Maximizing this log likelihood function results in estimates for m, q, p_1, and p_2 that are displayed in Figure A.1.2. These results are consistent with (a) full coverage of the birth cohort with NMI ($m = 1$) and (b) full temporary protection from RSV infection for infant with NMI ($q = 0$).

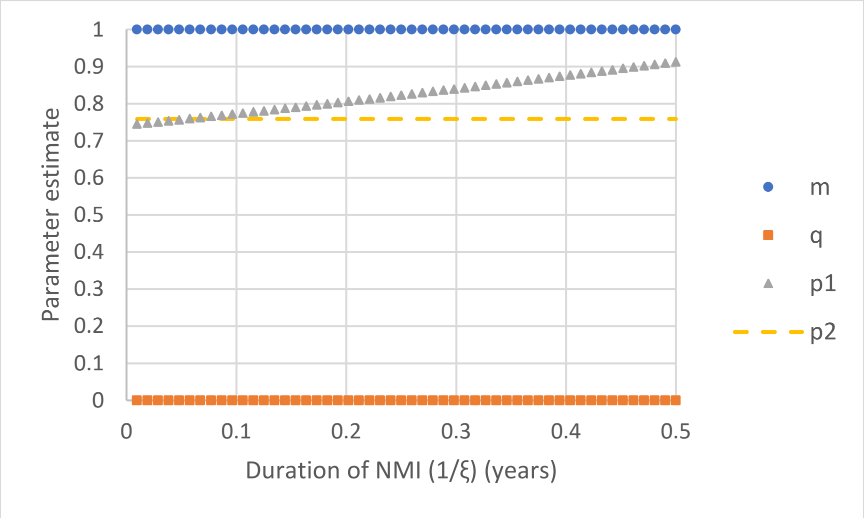

Supplemental Figure A.1.2: Parameter estimates m, q, p_1, and p_2 as a function of duration of NMI (ξ^{-1}). *(Blue dots) Probability of being born NMI (m). (Orange squares) Annualized probability of RSV naïve infants with NMI becoming infected with RSV (q). (Grey triangles) Annualized probability of RSV naïve < 2-year-olds becoming infected with RSV (p_1). (Yellow dashed line) Probability of previously infected 1-year-old becoming reinfected with RSV in their second year of life (p_2).*

Appendix A.2: Demographic model structure

We summarize stratification of population by age in Supplemental Table A.2.1 for a summary. Supplemental Table A.2.1 also characterizes ageing rates as either (a) inverse of the width of the age strata of origin (i.e., Inverse), (b) other aging schemes (i.e., Other), or (c) not applicable (i.e., N/A; for models integrated over only one RSV season). Finally, one model does not stratify the population by age, but does stratify the population by geographic location (i.e., stratification by state for a model of the United States).[14]

Supplemental Table A.2.1: Age stratification in RSV DTMs.

Model	Age strata	Ageing rates
Acedo, et al. (2010).[15] and<br>Acedo, Moraño, Díez-Domingo. (2010).[16]	- < 1-year-olds<br>- ≥ 1-year-olds	Other
Leecaster, et al. (2011).[17] and<br>Moore, et al. (2014).[18]	- < 2-year-olds<br>- ≥ 2-year-olds	Inverse
Kinyanjui, et al. (2015).[1]	- Monthly for < 2-year-olds<br>- Yearly for 2 – 77-year-olds<br>- ≥ 78-year-olds	Inverse
Pitzer, et al. (2015).[7]	- Monthly for < 1-year-olds<br>- 1 – 4-year-olds<br>- 5 – 9-year-olds<br>- 10 – 19-year-olds<br>- 20 – 39-year-olds<br>- 40 – 59-year-olds<br>- ≥ 60-year-olds	Inverse
Poletti, et al. (2015).[5,a]	- Unreported[b]	Not applicable
Hogan, et al. (2016).	- < 1-year-old<br>- 1-year-olds	Inverse
Yamin, et al. (2016).	- < 6-month-olds<br>- 6 – 11-month-olds<br>- 1-year-olds<br>- 2 – 4-year-olds<br>- 5 – 24-year-olds<br>- 25 – 49-year-olds<br>- 50 – 64-year-olds<br>- ≥ 65-year-olds	Other
Hogan, et al. (2017).[6]	- Monthly for < 5-year-olds<br>- 5-yearly for ≥ 5-year-olds	Inverse
Pan-Ngum, et al. (2017).[2]<br>(SAI model)	- Monthly for < 2-year-olds<br>- Yearly for 2 – 75-year-olds<br>- ≥ 76-year-olds	Inverse
Pan-Ngum, et al. (2017).[2]<br>(BWI model)	- Monthly for < 1-year-olds<br>- 2 – 5-year-olds<br>- 6 – 10-year-olds<br>- ≥ 11-year-olds	Inverse
Goldstein, et al. (2018).[19]	- < 3-year-olds<br>- 3 – 4-year-olds<br>- 5 – 6-year-olds<br>- 7 – 12-year-olds<br>- 13 – 19-year-olds<br>- 20 – 39-year-olds<br>- 40 – 59-year-olds<br>- ≥ 60-year-olds	Not applicable
Kombe, et al. (2019).[20,c]	- Unreported[b]	Not applicable
Arguedas, Santana-Cibrian, Velasco-Hernández. (2019)	- < 5-year-olds<br>- 5 – 19-year-olds<br>- 20 – 59-year-olds<br>- ≥ 60-year-olds	Inverse
**Continued next page.**		

[a] In addition to stratification by age, this model stratifies the population by household and primary school.
[b] Agent-based models do not report boundaries for age strata.
[c] In addition to stratification by age, these models stratify the population by household.

Supplemental Table A.2.1 (continued): Age stratification in RSV DTMs.

Model	Age strata	Ageing rates
Mahikul, et al. (2019).[21,c]	- < 2-year-olds<br>- 2 – 14-year-olds<br>- 15 – 59-year-olds<br>- ≥ 60-year-olds	Other
Brand, et al. (2020).[3,c]	- For households, individuals are sorted into ages:<br>- < 1-year-olds<br>- ≥ 1-year-olds<br>- For other model quantities (e.g., including community transmission and hospitalization), computations use age strata:<br>- Monthly for < 1-year-olds<br>- Yearly for 1 – 17-year-olds<br>- ≥ 18-year-olds	Inverse
Campbell, Geard, Hogan. (2020).[12,c]	- Exact.[b]	Other
Hodgson, et al. (2020).[9]	- Monthly for < 1-year-olds<br>- Yearly for 1 – 4-year-olds<br>- 5-yearly for 5 – 74-year-olds<br>- ≥ 75-year-olds	Inverse
Kinyanjui, et al. (2020).[4]	- Unreported<br>- See potentially<br>- Kinyanjui, et al. (2015),[1] or<br>- Pan-Ngum, et al. (2017).[2] (SAI model).	Inverse
van Boven, et al. (2020).[22]	- < 1-year-olds<br>- 1 – 4-year-olds<br>- 5 – 9-year-olds<br>- 10 – 19-year-olds<br>- 20 – 44-year-olds<br>- 45 – 64-year-olds<br>- ≥ 65-year-olds	Inverse

[a] In addition to stratification by age, this model stratifies the population by household and primary school.
[b] Agent-based models either do not report boundaries for age strata (Unreported) or they use exact age for agents (Exact).
[c] In addition to stratification by age, these models stratify the population by household.

Appendix A.3: Interventions

Representative results for interventions implemented in RSV DTMs are summarized in Supplemental Table A.3.1.

Supplemental Table A.3.1: Interventions implemented in RSV DTMs.

Model	Timing	Effective coverage[a] (%)	Duration (days)	Outcomes
**Maternal vaccination inducing partial temporary immunity for child only**				
Pan-Ngum, et al. (2017).[2]	- Birth	35	91	- 7 – 15% reduction in hospitalizations in < 1-year-olds
Hogan, et al. (2017).[6]	- Birth	40	183	- 6 – 37% reduction in hospitalizations in < 3-month-olds<br>- 30 – 46% reduction in hospitalizations in 3 – 5-month-olds
			91	- 25% reduction in hospitalizations in < 3-month-olds
**Maternal vaccination inducing full temporary immunity for child only**				
van Boven, et al. (2020).[22]	- Birth	50	183	- 26% reduction in infections in < 1-year-olds<br>- 13% increase in infections in 1 – 4-year-olds<br>- 4% increase in infections in 5 – 9-year-olds
**Continued next page.**				

N/A – Not applicable.
[a] Effective coverage is the product of coverage and effectiveness.
[b] Coverage varies by age: < 5-year-olds (80%), 5 – 24-year-olds (48%), 25 – 49-year-olds (33%), ≥ 50-year-olds (60%).
[c] Coverage of 50% of the population with a vaccine that reduces susceptibility by 50%.
[d] Awareness campaign reduces susceptibility of the entire population by 20%; equivalently, transmission (b_0) is reduced by 20%.

Supplemental Table A.3.1 (continued): Interventions implemented in RSV DTMs.

Model	Timing	Effective coverage[a] (%)	Duration (days)	Outcomes
**Maternal vaccination inducing partial temporary immunity for child only**				
Pan-Ngum, et al. (2017).[2]	- Birth	35	91	- 7 – 15% reduction in hospitalizations in < 1-year-olds
Hogan, et al. (2017).[6]	- Birth	40	183	- 6 – 37% reduction in hospitalizations in < 3-month-olds<br>- 30 – 46% reduction in hospitalizations in 3 – 5-month-olds
			91	- 25% reduction in hospitalizations in < 3-month-olds
**Maternal vaccination inducing full temporary immunity for child only**				
van Boven, et al. (2020).[22]	- Birth	50	183	- 26% reduction in infections in < 1-year-olds<br>- 13% increase in infections in 1 – 4-year-olds<br>- 4% increase in infections in 5 – 9-year-olds
				**Continued next page.**
**Maternal vaccination inducing full temporary immunity for both child and mother**				
Poletti, et al. (2015).[5]	- Birth	60	183	- 17% reduction in infections in < 1-year-olds<br>- 3% reduction in infections in the general population
Brand, et al. (2020).[3]	- Beginning of 3rd trimester	50	96	- 19% reduction in hospitalizations in < 5-year-olds
Hodgson, et al. (2020).[9]	- Beginning of 3rd trimester (Aug. – Dec.)	32	134	- 8.5% reduction in hospitalizations
**Maternal vaccination inducing partial temporary immunity for child and full temporary immunity for mother**				
Cambell, Geard, Hogan. (2020).[12]	- Beginning of 3rd trimester	N/A	90	- With coverage of 70% (effective coverage not reported): 16.6% reduction in infections for < 3-month-olds, 5.3% reduction in infections for 3 – 6-month-olds
**Vaccination inducing partial temporary immunity**				
Yamin, et al. (2016).[8]	- Annually with same timing as influenza vaccination (< 5-year-olds)	48	203	- 56% reduction in infections for < 5-year-olds<br>- 54% reduction in infections for ≥ 50-year-olds
	- Annually with same timing as influenza vaccination (entire population)	20 – 48[b]	203	- 65% reduction in infections for < 5-year-olds<br>- 75% reduction in infections for ≥ 50-year-olds
Pan-Ngum, et al. (2017).[2]	- 2 and 4 months	90	365	- 58 – 89% reduction in hospitalizations for < 1-year-olds
Smith, Hogan, Mercer. (2017).	- Annually at peak of RSV season	25[c]	730	- 35% reduction in infections in the general population
Kinyanjui, et al. (2020).[4]	- 2 and 4 months	90	365	- 51 – 88% reduction in hospitalizations in < 1-year-olds
**Vaccination inducing full temporary immunity**				
Acedo, et al. (2010).[15]	- Birth	85	365	- 75% reduction of infections in < 1-year-olds
Acedo, Moraño, Díez-Domingo. (2010).[16]	- 2, 4, and 12 months	85	Unreported	- 67% reduction in hospitalizations in < 1-year-olds
Kinyanjui, et al. (2015).[1]	- < 10-months	80	183	- 51 – 88% reduction in hospitalizations in < 6-month-olds
Poletti, et al. (2015).[5]	- 3 months	80	182	- 35% reduction in infections in < 1-year-olds
	- At primary school enrollment			- 32% reduction in infections in < 1-year-olds<br>- 36% reduction in infections in the general pop'n.
**Continued next page.**				

N/A – Not applicable.

[a] Effective coverage is the product of coverage and effectiveness.

[b] Coverage varies by age: < 5-year-olds (80%), 5 – 24-year-olds (48%), 25 – 49-year-olds (33%), ≥ 50-year-olds (60%).

[c] Coverage of 50% of the population with a vaccine that reduces susceptibility by 50%.

[d] Awareness campaign reduces susceptibility of the entire population by 20%; equivalently, transmission (b_0) is reduced by 20%.

Supplemental Table A.3.1 (continued): Interventions implemented in RSV DTMs.

Model	Timing	Effective coverage[a] (%)	Duration (days)	Outcomes
**Vaccination inducing full temporary immunity (continued)**				
Jornet-Sanz, et al. (2017).[23]	- Birth	80	183	- 81% reduction in hospitalizations of < 2-year-olds
Nugraha, Nuraini. (2017).[24]	- Birth	2	203	- 21% reduction in infections in the general population
Goldstein, et al. (2018).[19]	- Annually prior to RSV season	Unreported	Unreported	- Vaccination of 3 – 6-year-olds results in the greatest reduction in the initial effective reproduction number
Hodgson, et al. (2020).[9]	- 2 months	75	359	- 6.8% reduction in hospitalizations
	- Annually (Oct. – Feb.) for 2 – 4-year olds	37		- 3.6% reduction in hospitalizations
	- Annually (Oct. – Feb.) for 5 – 9-year-olds	50		- 2.1% reduction in hospitalizations
	- Annually (Aug. – Dec.) for 5 – 14-year-olds	50		- 4.8% reduction in hospitalizations
	- Anually (Nov. – Mar.) for ≥ 65-year-olds	58		- 28.0% reduction in hospitalizations
	- Anually (Nov. – Mar.) for ≥ 75-year-olds	58		- 21.9% reduction in hospitalizations
van Boven, et al. (2020).[22]	- < 6-month-olds	50	1,642.5	- 30% reduction in infections in < 1-year-olds<br>- 21% reduction in infections in 1 – 4-year-olds<br>- 8% reduction in infections in 5 – 9-year-olds
**Monoclonal antibody immunoprophylaxis inducing full temporary immunity**				
Hodgson, et al. (2020).[9]	- At birth (born in-season; Oct. – Feb.) or beginning of season (born out-of-season) for infants born at < 34 weeks gestational age with CHD or CLD and < 9-months-old at beginning of season	30	150	- 0.2% reduction in hospitalizations
	- At birth (born in-season; Oct. – Feb.) or beginning of season (born out-of-season) for infants born at < 34 weeks gestational age with CHD or CLD and < 9-months-old at beginning of season	63	250	- 0.3% reduction in hospitalizations
	- At birth (born in-season; Oct. – Feb.) or beginning of season (born out-of-season) all infants	63	250	- 7.9% reduction in hospitalizations
**Continued next page.**				

N/A – Not applicable.

[a] Effective coverage is the product of coverage and effectiveness.

[b] Coverage varies by age: < 5-year-olds (80%), 5 – 24-year-olds (48%), 25 – 49-year-olds (33%), ≥ 50-year-olds (60%).

[c] Coverage of 50% of the population with a vaccine that reduces susceptibility by 50%.

[d] Awareness campaign reduces susceptibility of the entire population by 20%; equivalently, transmission (b_0) is reduced by 20%.

Supplemental Table A.3.1 (continued): Interventions implemented in RSV DTMs.

Model	Timing	Effective coverage[a] (%)	Duration (days)	Outcomes
**Maternal vaccination (inducing full temporary immunity for mother and child) and household vaccination inducing full temporary immunity**				
Brand, et al. (2020).[3]	- Maternal vaccination at beginning of 3rd trimester<br>- Household vaccination at birth	75	96<br><br>183	- 50% reduction in hospitalizations for < 5-year-olds
**Awareness campaign reducing susceptibility**				
Nugraha, Nuraini. (2017).[24]	- Continuously through the year	20[d]	N/A	- 38% reduction in infections in the general population
**Vaccination inducing full temporary immunity and awareness campaign reducing susceptibility**				
Nugraha, Nuraini. (2017).[24]	- Vaccine at birth from start of season to peak in RSV incidence<br>- Awareness campaign continuously throughout the year	2<br><br>20[d]	203<br><br>N/A	- 56% reduction in infections in the general population
**Treatment**				
Rosa, Torres. (2018)a.[25]	- Continuously throughout the year	N/A	N/A	- Model formulates and solves an optimal control problem for an *SEIRS* ODE model.
Rosa, Torres. (2018)b.[26]	- Continuously throughout the year	N/A	N/A	- Model formulates and solves and optimal control problem for an *SEIRS* FDE model.

N/A – Not applicable.
[a] Effective coverage is the product of coverage and effectiveness.
[b] Coverage varies by age: < 5-year-olds (80%), 5 – 24-year-olds (48%), 25 – 49-year-olds (33%), ≥ 50-year-olds (60%).
[c] Coverage of 50% of the population with a vaccine that reduces susceptibility by 50%.
[d] Awareness campaign reduces susceptibility of the entire population by 20%; equivalently, transmission (b_0) is reduced by 20%.

Appendix A.4: Calibration data

Supplemental Table A.4.1 summarizes RSV epidemic data sets used in model calibration for RSV DTMs. We remark that three types of data are differentiated: in-patient data (i.e., hospitalizations), in-patient and outpatient data (i.e., detections), and Google searches for the term "RSV".

Supplemental Table A.4.1: RSV epidemic data used in calibration of RSV DTMs.

Location	Type (Age range)	Time period (Frequency)	Model	References	Notes
**Australia**					
Perth	Hospitalizations (< 2-year-olds)	2000 – 2005 (Weekly)	- Moore, et al. (2014).[18]<br>- Hogan, et al. (2016).[27]		
	Hospitalizations (< 2-year-olds)	2000 – 2013 (Monthly)	- Hogan, et al. (2017).[6]		
	Other (< 1-year-olds)	N/A	- Campbell, Geard, Hogan. (2020).[12]	- Hall. (1981).[28]<br>- Glezen, et al. (1986).[13]<br>- Hogan, et al. (2016).[29]<br>- Jacoby, Glass, Moore. (2016).[30]	Transmission parameters are chosen from Hogan, et al. (2017).[6] Other transmission parameters are chosen to reproduce annual or biennial peaks in RSV incidence, proportion of infant RSV infections caused by older siblings, and proportion of infants infected in their first year of life.
**Brazil**					
Porto Alegre	Detections (< 5-year-olds)	1990 – 2003 (Monthly)	- White, et al. (2007).[31]	- Straliotto, Nestor, Siqueira. (2001).[32]	
Rio de Janeiro	Detections (< 5-year-olds)	1986 – 2006 (Monthly)	- White, et al. (2007).[31]	- Siqueira, Nascimento, Anderson. (1991).[33]<br>- Nascimento, et al. (1991).[34]	
**Colombia**					
Bogotá	Detections (< 5-year-olds)	2005 – 2010 (Weekly)	- Aranda-Lozano, González-Para, Jódar. (2013).[35]		Data were collected by the surveillance system Sistema Integrado de Información para la Vigilancia de la Salud Pública (SIVIGLIA). All data were recorded in the Sistema de Información de Labotorio de Salud Pública (SILASP) public health laboratory database.
**Continued next page.**					

Supplemental Table A.4.1 (continued): RSV epidemic data used in calibration of RSV DTMs.

Location	Type (Age range)	Time period (Frequency)	Model	References	Notes
**Finland**					
Turku	Hospitalizations (< 10-year-olds)	1980 – 2001 (Weekly)	- Weber, Weber, Milligan. (2001).[10]<br>- White, et al. (2005).[36]<br>- White, et al. (2007).[31]<br>- Ponciano, Capistrán. (2011).[37]	- Waris. (1991).[38]	Monthly proportion of RSV detections that are RSV group A are available.
**The Gambia**					
The Gambia	Detections (< 2-year-olds)	1990 – 1994 (Monthly)	- Weber, Weber, Milligan. (2001).[10]<br>- White, et al. (2007).[31]<br>- Ponciano, Capistrán. (2011).[37]	- Weber, et al. (1998).[39]<br>- Cane, et al. (1999).[40]	
**Kenya**					
Kilifi	Detections (< 3-year-olds)	2002 – 2005 (Weekly)	- Mwambi, et al. (2011).[41]<br>- Poletti, et al. (2015).[5]	- Nokes, et al. (2004).[42]<br>- Nokes, et al. (2008).[43]<br>- Ohuma, et al. (2012).[44]	
	Hospitalizations (< 5-year-olds)	2004 – 2010 (Monthly)	- Kinyanjui, et al. (2015).[1]<br>- Pan-Ngum, et al. (2017).[2]	- Nokes, et al. (2009).[45]	
	Detections (All ages)	2009 – 2010 (Biweekly)	- Kombe, et al. (2019).[20]	- Munywoki. (2013).[46]<br>- Munywoki, et al. (2014).[47]<br>- Munywoki, et al. (2015)a.[48]<br>- Munywoki, et al. (2015)b.[49]	
	Hospitalizations (< 5-year-olds)	2001 – 2016 (Weekly)	- Brand, et al. (2020).[3]	- Nokes, et al. (2009).[45]	
**Mexico**					
San Luis Potosi	Detections (All ages)	2000 – 2010 (Weekly)	- Arguedas, Sandana-Cibrian, Velasco-Hernández. (2019).[50]		Data are reported by the State Department of Epidemiology and Health Services
Various states	Hospitalizations (All ages)	2000 – 2014 (Weekly)	- Baker, et al. (2019).[51]		Data are reported in the Subsistema Automatizado de Egresos Hospitalarios by the Dirección General de Informacion en Sauld.
**The Netherlands**					
The Netherlands	Detections (All ages)	2013 – 2017 (Weekly)	- van Boven, et al. (2020).[22]	- Vos, et al. (2019).[52]	Data are reported by the National Institute for Public Health and the Environment (RIVM)/Nivel sentinel surveillance of influenza-like illness (ILI) and acute respiratory illness (ARI). Data are age stratified.
	Hospitalizations (All ages)	2013 – 2017 (Weekly)			Data are reported by the Dutch Hospitalization Data (DHD) organization. Data are age stratified.
**Continued next page.**					

Supplemental Table A.4.1 (continued): RSV epidemic data used in calibration of RSV DTMs.

Location	Type (Age range)	Time period (Frequency)	Model	References	Notes
**The Netherlands (continued)**					
The Netherlands	General practice consultations (All ages)	2013 – 2017 (Weekly)	- van Boven, et al. (2020).[22]		Data are reported by the Nivel Primary Care Database
**Philippines**					
Bohol	Detections (< 2-year-olds)	2000 – 2004 (Weekly)	- Paynter, et al. (2014).[53]<br>- Paynter. (2016).[54]	- Lucero, et al. (2009).[55]<br>- Simões, et al. (2013).[56]	
**Singapore**					
Singapore	Detections (All ages)	1990 – 1995 (Monthly)	- Weber, Weber, Milligan. (2001).[10]<br>- White, et al. (2007).[31]	- Chew, et al. (1998).[57]	
**Spain**					
Madrid	Hospitalizations (< 2-year-olds)	1990 – 2002 (Monthly)	- White, et al. (2007).[31]	- Garcia, et al. (2001).[58]	
Valencia	Hospitalizations (< 4-year-olds)	2001 – 2005 (Monthly)	- Arenas, González-Parra, Moraño. (2009).[59]<br>- Arenas, González-Parra, Jódar. (2010).[60]		Data from CMBD (basic minimum database) of the Spanish region of Valencia.
	Hospitalizations (< 1-year-olds)	2001 – 2004 (Weekly)	- Acedo, et al. (2010).[15]<br>- Acedo, Moranõ, Díez-Domingo. (2010).[16]<br>- Corberán-Vallet, Santonja. (2014).[61]<br>- Jornet-Sanz, et al. (2017).[23]		Data from CMBD (basic minimum database) of the Spanish region of Valencia.
**Thailand**					
Sa Kaeo and Nakhon Phanom	Hospitalizations (All ages)	2004 – 2011 (Monthly)	- Mahikul, et al. (2019).[21]	- Fry, et al. (2010).[62]<br>- Naorat, et al. (2013).[63]	
**United Kingdom**					
Birmingham	Detections (< 1-year-olds)	1989 – 2001 (Annual)	- White, et al. (2005).[36]	- Cane, et al. (1994).[64]	Annual proportion of RSV detections that are group A.
England & Wales	Hospitalizations (Unreported)	1991 – 2000 (Weekly)	- White, et al. (2005).[36]<br>- White, et al. (2007).[31]		Data from Communicable Disease Surveillance Centre, UK.
	Hospitalizations (≤ 5-year-olds)	2000 – 2013 (Weekly)	- Kinyanjui, et al. (2020).[4]		Data from Public Health England (PHE).
England	Detections (< 5-year-olds, 5 – 14-year-olds, 15 – 44-year-olds, 45 – 64-year-olds, ≥ 65-year-olds)	2010-2017 (Weekly)	- Hodgson, et al. (2020).[9]	- Zhao, et al. (2014).[65]	Respiratory DataMart System from Public Health England and the National Health Service.
**Continued next page.**					

Supplemental Table A.4.1 (continued): RSV epidemic data used in calibration of RSV DTMs.

**Location**	**Type (Age range)**	**Time period (Frequency)**	**Model**	**References**	**Notes**
**United Kingdom (continued)**					
West Midlands	Unreported (Unreported)	1991 – 1998 (Weekly)	- White, et al. (2007).[31]		Data from Health Protection Agency (West Midlands), Communicable Disease Surveillance Centre, UK.
**United States**					
Florida	Detections (Unreported)	1981 – 1997 (Monthly)	- Weber, Weber, Milligan. (2001).[10]<br>- White, et al. (2007).[31]	- Halstead, Jenkins. (1998).[66]	
	Detections (Unreported)	2011 – 2014 (Monthly)	- Rosa, Torres. (2018)a.[25]<br>- Rosa, Torres. (2018)b.[26]		Data from Florida Department of Health, Respiratory Syncytial Virus (RSV) in Florida.
North Carolina	Detections (Children)	2003 – 2006 (Monthly)	- Nugraha, Nuraini. (2017).[24]	- Wilfret, et al. (2008).[67]	
Salt Lake City, Utah	Detections (Children)	2001 – 2008 (Daily)	- Leecaster, et al. (2011).[17]		
Various states	Hospitalizations (All)	1989 – 2009 (Weekly)	- Pitzer, et al. (2015).[7]		Data from the Healthcare Cost and Utilization Project, State Inpatient Database.
	Detections (All)	2004 – 2014 (Weekly)	- Reis, Shaman. (2016).[68]<br>- Reis, Shaman. (2018).[69]		US Data from Centers for Disease Control and Prevention, National Respiratory and Enteric Virus Surveillance System. Data are given by census division and Health and Human Services region.
	Detections (All)	2010 – 2014 (Weekly)	- Yamin, et al. (2016).[8]		US Data from Centers for Disease Control and Prevention, National Respiratory and Enteric Virus Surveillance System. State data are used for California, Colorado, Pennsylvania, and Texas.
	Hospitalizations (All ages)	2001 – 2012 (Annual)	- Goldstein, et al. (2018).[19]		Data from the Healthcare Cost and Utilization Project, State Inpatient Database.
	Google search (N/A)	2013 – 2018 (Weekly)	- Seroussi, et al. (2020).[14]	- Oren, et al. (2018).[70]	Data are given for all states.
	Hospitalizations (All)	1997 – 2011 (Weekly)	- Baker, et al. (2019).[51]		Data from the Healthcare Cost and Utilization Project, State Inpatient Database.

Appendix A.5: Common parameter values

Common parameter values determined through literature search and model calibration are reported in Supplemental Table A.5.1-Supplemental Table A.5.7. We remark that Supplemental Table A.5.7 reports parameterization results for a set of parameters not discussed in the main text: the social mixing matrix (C). The social mixing matrix measures the strength of interactions between different age strata with respect to the transmission of RSV. Because of the complexity of social mixing matrices, we do not report values for social mixing matrices. Instead, we report the models that use social mixing matrices and the references to literature used to construct social mixing matrices.

Supplemental Table A.5.1: Parameterization of the natural maternal immunity waning rate (ξ) in RSV DTMs.

Model	Rate (per year)	Duration (days)	Reference
**Literature values**			
- Weber, Weber, Milligan. (2001).[10]<br>- Arenas, González-Parra, Moraño. (2009).[59]	13.00	28.1	- Ogilvie, et al. (1981).[71]
- Pitzer, et al. (2015).[7]	3.25	112.3	- Ochola, et al. (2009).[72]
- Poletti, et al. (2015).[5]	3.00	121.7	- Ochola, et al. (2009).[72]
- Yamin, et al. (2016).[8]	3.44	106.1	- Ochola, et al. (2009).[72]
- Campbell, Geard, Hogan. (2020).[12]	4.06	90.0	- Assumption
- Hodgson, et al. (2020).[9]	2.73	133.5	- Glezen, et al. (1981).[73]<br>- Ogilvie, et al. (1981).[71]<br>- Ochola, et al. (2009).
**Calibrated values**			
- Kinyanjui, et al. (2015).[1]	5.22	69.9	Calibrated value
- Pan-Ngum, et al. (2017).[2] (SAI model)	5.92	61.7	Calibrated value
- Pan-Ngum, et al. (2017).[2] (BWI model)	40.11	9.1	Calibrated value
- Brand, et al. (2020).[3]	16.89	21.6	Calibrated value
- Kinyanjui, et al. (2020).[4] (SAI model)	12.00	30.4	Calibrated value
- Kinyanjui, et al. (2020).[4] (BWI model)	49.58	7.4	Calibrated value

Supplemental Table A.5.2: Parameterization of relative susceptibility to RSV infection (τ) in RSV DTMs.

Model	Symbol	Description	Value	Reference
**Stratification by age**				
- Moore, et al. (2014).[18]	$\tau_{<2}$	< 2-year-olds	1.000[a]	- Henderson, et al. (1979).[74]<br>- Hall. (1981).[28]
	$\tau_{\geq 2}$	≥ 2-year-olds	0.650	
- Hogan, et al. (2017).[6]	τ	> 3-month-olds	1.000[a]	- Cox, et al. (1998).[75]
	$\tau_{<1}$	< 1-month-olds	0.080	
	τ_{1-2}	1 – 2-month-olds	0.450	
- Goldstein, et al. (2018).[19,b]	τ	Various		- Assumption
- Hogan, et al. (2016).[27]	$\tau_{<1}$	< 1-year-old	1.000[a]	- Calibrated value
	τ_1	1-year-old	0.228	
- Yamin, et al. (2016).[8]	τ_0	RSV naïve individuals	1.000[a]	- Calibrated values
	$\tau_{<2}$	< 2-year-olds	3.074 – 3.940[c]	
	τ_{2-4}	2 – 4-year-olds	0.521 – 1.053[c]	
	τ_{5-49}	5 – 49-year-olds	0.050 – 0.088[c]	
	$\tau_{\geq 50}$	≥ 50-year-olds	0.120 – 0.250[c]	
- Arguedas, Santana-Cibrian, Velasco-Hernández. (2019).[50]	τ_{0-4}	0 – 4-year-olds	1.000[a]	- Calibrated values
	τ_{5-19}	5 – 19-year-olds	0.240	
	τ_{20-59}	20 – 59-year-olds	0.060	
	$\tau_{\geq 60}$	≥ 60-year-olds	0.240	
**Stratification by infection history**				
- Weber, Weber, Milligan. (2001).[10]	τ_1	RSV naïve	1.000[a]	- Assumptions
	τ_2	1 previous RSV infection	0.500	
	τ_3	2 previous RSV infections	0.350	
	τ_4	≥ 3 previous RSV infections	0.250	
- Paynter, et al. (2014).[53]	τ_1	RSV naïve	1.000[a]	- Kapikian, et al. (1961).[76]<br>- Kravetz, et al. (1961).[77]<br>- Mills, et al. (1971).[78]<br>- Henderson, et al. (1979).[74]<br>- Glezen, et al. (1986).[13]<br>- Watt, et al. (1990).[79]<br>- DeVincenzo, et al. (2010).[80]<br>- Ohuma, et al. (2012).[44]
	τ_2	≥ 1 previous RSV infections	0.770	
- Kinyanjui, et al. (2015).[1]<br>- Pan-Ngum, et al. (2017).[2] (SAI model)<br>- Kinyanjui, et al. (2020).[4] (SAI model)	τ_1	RSV naïve	1.000[a]	- Henderson, et al. (1979).[74]
	τ_2	1 previous RSV infection	0.750	
	τ_3	≥ 2 previous RSV infections	0.650	
- Morris, et al. (2015).[81]	τ_1	RSV naïve	1.000[a]	- Henderson, et al. (1979).[74]
	τ_2	≥ 1 previous RSV infections	0.450	
- Pitzer, et al. (2015).[7]	τ_1	RSV naïve	1.000[a]	- Monto, et al.(1974).[82]<br>- Hall, et al.(1976).[83]<br>- Henderson, et al. (1979).[74]<br>- Glezen, et al. (1986).[13]
	τ_2	1 previous RSV infection	0.760	
	τ_3	2 previous RSV infections	0.600	
	τ_4	≥ 3 previous RSV infections	0.400	
- Pan-Ngum, et al. (2017).[2] (BWI model)<br>- Mahikul, et al. (2019).[21]	τ_1	RSV naïve	1.000[a]	- Henderson, et al. (1979).[74]
	τ_2	≥ 1 previous RSV infections	0.540	
**Continued next page.**				

[a] Reference value.
[b] A full description of the non-standard method employed by Goldstein, et al. (2018).[19] is beyond the scope of this manuscript.
[c] Values vary by geographic area (i.e., by US state: California, Colorado, Pennsylvania, Texas).
[d] Values for other models are reported; we report values from the "best" performing model.
[e] Susceptibilities by age and infection history are multiplicative, e.g., susceptibility for age range 1 – 4-year-olds to homologous reinfection with RSV is $\tau_{1-4} \times \tau_{ho}$.

Supplemental Table A.5.2 (continued): Parameterization of relative susceptibility to RSV infection (τ) in RSV DTMs.

Model	Symbol	Description	Value	Reference
**Stratification by infection history (continued)**				
- Brand, et al. (2020).[3]	τ_1	RSV naïve	1.000[a]	- Henderson, et al. (1979).[74]
	τ_2	≥ 1 previous RSV infections	0.750	
- Hodgson, et al. (2020).[9]	τ_1	RSV naïve	1.000[a]	- Henderson, et al. (1979).[74]
	τ_2	1 previous RSV infection	0.890	
	τ_3	2 previous RSV infections	0.721	
	τ_4	≥ 3 previous RSV infections	0.238	
- Kinyanjui, et al. (2020).[4] (BWI model)	τ_1	RSV naïve	1.000[a]	- Henderson, et al. (1979).[74]
	τ_2	≥ 1 previous RSV infections	0.528	
- White, et al. (2005).[36]	τ_1	RSV naïve	1.000[a]	- Calibrated values
	τ_{ho}	Susceptibility to homologous reinfection	0.357	
	τ_{he}	Susceptibility to heterologous reinfection	0.843	
- White, et al. (2007).[31,d]	τ_1	RSV naïve	1.000[a]	- Calibrated value
	τ_2	≥ 1 previous RSV infections	0.680	
- Poletti, et al. (2015).[5]	τ_1	RSV naïve	1.000[a]	- Calibrated value
	τ_2	≥ 1 previous RSV infections	0.880	
**Stratification by age and RSV infection history**				
- Kombe, et al. (2019).[20,e]	$\tau_{<1}$	RSV naïve	1.000[a]	- Calibrated values
	τ_{1-4}	1 – 4-year-olds	0.930	
	τ_{5-14}	5 – 14-year-olds	0.480	
	$\tau_{\geq 15}$	≥ 15-year-olds	0.430	
	τ_{ho}	Susceptibility to homologous reinfection	0.630	
	τ_{he}	Susceptibility to heterologous reinfection	0.680	
**Stratification by maternal immunity type**				
- Campbell, Geard, Hogan. (2020).[12]	τ	No maternal immunity	1.000[a]	- Assumption
	τ_V	Maternal immunity from vaccinated mothers	0.400	
	τ_I	Natural maternal immunity	0.400	
**Stratification by nutritional status**				
- Paynter. (2016).[54]	τ_W	Well-nourished	1.000[a]	- Calibrated value depends on degree of mixing between well-nourished and malnourished children.
	τ_M	Malnourished	1.1 – 1.4	

[a] Reference value.
[b] A full description of the non-standard method employed by Goldstein, et al. (2018).[19] is beyond the scope of this manuscript.
[c] Values vary by geographic area (i.e., by US state: California, Colorado, Pennsylvania, Texas).
[d] Values for other models are reported; we report values from the "best" performing model.
[e] Susceptibilities by age and infection history are multiplicative, e.g., susceptibility for age range 1 – 4-year-olds to homologous reinfection with RSV is $\tau_{1-4} \times \tau_{ho}$.

Supplemental Table A.5.3: Parameterization of relative infectiousness to RSV infection (η) in RSV DTMs.

Model	Symbol	Description	Value	Reference
**Stratification by age**				
- Moore, et al. (2014).[18]	$\eta_{<2}$	< 2-year-olds	1.000[a]	- Assumption
	$\eta_{\geq 2}$	≥ 2-year-olds	0.650	
- Hogan, et al. (2016).[27]	$\eta_{<1}$	< 1-year-olds	1.000[a]	- Assumption
	η_{1}	1-year-olds	1.000	
- Hogan, et al. (2017).[6]	$\eta_{<10}$	< 10-year-olds	1.000[a]	- Assumption
	$\eta_{\geq 10}$	≥ 10-year-olds	0.600	
- Arguedas, Santana-Cibrian, Velasco-Hernández. (2019).[50]	η_{0-4}	0 – 4-year-olds	1.000[a]	- Assumption
	η_{5-19}	5 – 19-year-olds	1.000	
	η_{20-59}	20 – 59-year-olds	1.000	
	$\eta_{\geq 60}$	≥ 60-year-olds	1.000	
- Campbell, Geard, Hogan. (2020).[12]	$\eta_{<10}$	< 10-year-olds	1.000[a]	- Calibrated value
	$\eta_{\geq 10}$	≥ 10-year-olds	0.200	
**Stratification by infection history**				
- Weber, Weber, Milligan. (2001).[10]	η_{1}	RSV naïve	1.000[a]	- Assumption
	η_{2}	1 previous RSV infection	1.000	
	η_{3}	2 previous RSV infections	1.000	
	η_{4}	≥ 3 previous RSV infections	1.000	
- Paynter, et al. (2014).[53]	η_{1}	RSV naïve	1.000[a]	- Hall, Douglas, Geiman. (1976).[84] - Hall, et al. (1991).[85] - Hall, et al. (2001).[86]
	η_{2}	≥ 1 previous RSV infections	0.700	
- Kinyanjui, et al. (2015).[1] - Pan-Ngum, et al. (2017).[2] (SAI model). - Kinyanjui, et al. (2020).[4] (SAI model).	η_{1}	RSV naïve	1.000[a]	- Assumption
	η_{2}	1 previous RSV infection	0.500	
	η_{3}	≥ 2 previous RSV infections	0.250	
- Morris, et al. (2015).[81]	η_{1}	RSV naïve	1.000[a]	- Henderson, et al. (1979).[74] - Hall, et al. (1991).[85]
	η_{2}	≥ 1 previous RSV infections	0.250	
- Pitzer, et al. (2015).[7]	η_{1}	RSV naïve	1.000[a]	- Henderson, et al. (1979).[74] - Glezen, et al. (1986).[13] - Nokes, et al. (2008).[43]
	η_{2}	1 previous RSV infection	0.750	
	η_{3}	≥ 2 previous RSV infections	0.510	
- Brand, et al. (2020).[3]	η_{1}	RSV naïve	1.000[a]	- Kinyanjui, et al. (2015).[1]
	η_{2}	≥ 1 previous RSV infections	0.500[b]	
- White, et al. (2005).[36]	η_{1}	RSV naïve	1.000[a]	- Calibrated value
	η_{2}	≥ 1 previous RSV infections	0.413	
- White, et al. (2007).[31,c]	η_{1}	RSV naïve	1.000[a]	- Calibrated value
	η_{2}	≥ 1 previous RSV infections	0.600	
**Continued next page.**				

URTI – Upper respiratory tract infection; LRTI – Lower respiratory tract infection; SLRTI – Severe lower respiratory tract infection.
[a] Reference value.
[b] Calibrated value.
[c] Values for other models are reported; we report values from the "best" performing model.
[d] A full description of the non-standard methods employed by Yamin, et al. (2016).[8] and Kombe, et al. (2018).[20] are beyond the scope of this manuscript.
[e] Infectiousness values reported here are multiplicative, e.g., infectiousness for a symptomatic individual infected with RSV group A with low viral load in a large household is $\eta_A \times \eta_{HH} \times \eta_{LS}$.

Supplemental Table A.5.3 (continued): Parameterization of relative infectiousness to RSV infection (η) in RSV DTMs.

Model	Symbol	Description	Value	Reference
**Stratification by severity of RSV infection**				
- Pan-Ngum, et al. (2017).[2] (BWI model)<br>- Mahikul, et al. (2019).[21]	η_A	Asymptotic	0.200	- Values are calibrated in Pan-Ngum, et al. (2017).[2]<br>- Mahikul, et al. (2019).[21] references Pan-Ngum, et al. (2017).[2]
	η_U	URTI	0.450	
	η_L	LRTI	0.720	
	η_S	SLRTI	1.000[a]	
- Kinyanjui, et al. (2020).[4] (BWI model)	η_A	Asymptotic	0.177	- Pan-Ngum, et al. (2017).[2]
	η_U	URTI	0.467	
	η_L	LRTI	0.749	
	η_S	SLRTI	1.000[a]	
- Hodgson, et al. (2020).[9]	η_S	Symptomatic	1.000[a]	- Calibrated
	η_A	Asymptomatic	0.634	
**Stratification by multiple factors**				
- Poletti, et al. (2015).[5]	η_H	Household	1.000[a]	- Assumption
	η_S	School	1.000	
	η_C	Community	1.000	
- Yamin, et al. (2016).[8,d]	η	Various		- Hall, Douglas, Geiman. (1976).[84]<br>- DeVincenzo, et al. (2010).[80]<br>- Fairchok, et al. (2010).[87]
- Kombe, et al. (2018).[20,d,e]	η_1	Asymptomatic, low viral load, and small household	1.000[a]	- Calibrated values
	η_{LS}	Symptomatic, low viral load, and small household	0.070	
	η_{HA}	Asymptomatic, high viral load, and small household	2.480	
	η_{HS}	Symptomatic, high viral load, and small household	6.700	
	η_{HH}	Large household	0.424	
	η_A	RSV group A	0.019	
	η_B	RSV group B	0.015	

URTI – Upper respiratory tract infection; LRTI – Lower respiratory tract infection; SLRTI – Severe lower respiratory tract infection.
[a] Reference value.
[b] Calibrated value.
[c] Values for other models are reported; we report values from the "best" performing model.
[d] A full description of the non-standard methods employed by Yamin, et al. (2016).[8] and Kombe, et al. (2018).[20] are beyond the scope of this manuscript.
[e] Infectiousness values reported here are multiplicative, e.g., infectiousness for a symptomatic individual infected with RSV group A with low viral load in a large household is $\eta_A \times \eta_{HH} \times \eta_{LS}$.

Supplemental Table A.5.4: Parameterization of rate for emergence of infectiousness (σ) in RSV DTMs.

Model	Rate (per year)	Duration (days)	Reference
- Weber, Weber, Milligan. (2001).[10]<br>- Arenas, González-Parra, Moraño. (2009).[59]<br>- Rosa, Torres. (2018)a.[25]<br>- Rosa, Torres. (2018)b.[26]	91.00	4.01	- Kravetz, et al. (1961).[77]<br>- Ditchburn, et al. (1971).[88]
- Leecaster, et al. (2011).[17]<br>- Paynter. (2016).[54]	73.00	5.00	- Crowcroft, et al. (2008).[89]<br>- Heymann. (2008).[90]
- Moore, et al. (2014).[18]<br>- Hogan, et al. (2016).[27]<br>- Hogan, et al. (2017).[6]<br>- Campbell, Geard, Hogan. (2020).[12]	91.25	4.00	- Kravetz, et al. (1961).[77]<br>- Ditchburn, et al. (1971).[88]<br>- Lessler, et al. (2009).[91]
- Paynter, et al. (2014).[53]	60.83 – 91.25	4.00 – 6.00	- Kravetz, et al. (1961).[77]<br>- Hall, et al. (1976).[83]<br>- Hawker, et al. (2005).[92]<br>- Crowcroft, et al. (2008).[89]<br>- DeVincenzo, et al. (2010).[80]
- Arguedas, Santana-Cibrian, Velasco-Hernánzez. (2019).[50]	52.14	7.00	- Assumption
- Hodgson, et al. (2020).[9]	73.29	4.98	- DeVincenzo, et al. (2010).[80]

Model	Probability	Duration (days)	Reference
- Kombe, et al. (2019).[20]	1/3	2.00	- Lee, et al. (2004).[93]
	1/3	3.00	
	1/4	4.00	
	1/6	5.00	

Supplemental Table A.5.5: Parameterization of the recovery rate (ν) in RSV DTMs.

Model	Symbol	Description	Rate (per year)	Duration (days)	Reference
**Unstratified (recovery rate applied uniformly to entire population)**					
- Weber, Weber, Milligan. (2001).[10]<br>- Arenas, González-Parra, Moraño. (2009).[59]<br>- Arenas, González-Parra, Jódar. (2010).[60]<br>- Ponciano, Capistrán. (2011).[37]<br>- Aranda-Lozano, González-Parra, Querales. (2013).[35]<br>- Nugraha, Nuraini. (2017).[24]<br>- Smith, Hogan, Mercer. (2017).[11]<br>- Rosa, Torres. (2018)a.[25]<br>- Rosa, Torres, (2018)b.[26]	ν	Recovery rate	36.00	10.1	- Hall, Douglas, Geiman. (1976).[84]
- White, et al. (2005).[36]<br>- White, et al. (2007).[31]<br>- Arenas, González, Jódar. (2008).[94]<br>- Hogan, et al. (2016).[27]<br>- Hogan, et al. (2017).[6]<br>- Campbell, Geard, Hogan. (2020).[12]	ν	Recovery rate	40.56	9.0	- Hall, Douglas, Geiman. (1976).[84]<br>- Collins, et al. (1996).[95]<br>- Hall. (2004).[96]
- Acedo, et al. (2010).[15]<br>- Acedo, Moraño, Díez-Domingo. (2010).[16]<br>- Leecaster, et al. (2011).[17]<br>- Moore, et al. (2014).[18]<br>- Corberán-Vallet, Santonja. (2014).[61]<br>- Jornet-Sanz, et al. (2017).[23]	ν	Recovery rate	36.50	10.0	- Hall, Douglas, Geiman. (1976).[84]<br>- Hall. (2004).[96]
- Morris, et al. (2015).[81]	ν	Recovery rate	13.00	28.1	- Assumption
- Poletti, et al. (2015).[5]	ν	Recovery rate	33.18	11.0	- Munywoki, et al. (2015)b.[49]
- Baker, et al. (2019).[51,a]	ν	Recovery rate	26.07	14.0	- Assumption
- Reis, Shaman. (2016).[68]	ν	Recovery rate	57.03	6.4	- Calibrated value
- Goldstein, et al. (2018).[19]	ν	Recovery rate	46.80	7.8	- Crowcroft, et al. (2008).[89]
- Reis, Shaman. (2018).[69]	ν	Recovery rate	70.19	5.2	- Calibrated value
- Seroussi, Levy, Yom-Tov. (2020).[14]	ν	Recovery rate	1.04	351	- Calibrated value
- van Boven, et al. (2020).[22]	ν	Recovery rate	20.86	17.5	- Calibrated value
**Continued on next page.**					

URTI – Upper respiratory tract infection; LRTI – Lower respiratory tract infection; SLRTI – Severe lower respiratory tract infection.
[a] The modelling approach taken assumes that the time from infection to recovery is approximately two weeks. Movement from infectious to recovered compartment is not explicitly modelled.

Supplemental Table A.5.5 (continued): Parameterization of the recovery rate (ν) in RSV DTMs.

Model	Symbol	Description	Rate (per year)	Duration (days)	Reference
**Stratification by infection history**					
- Paynter, et al. (2014).[53]	ν_1	RSV naïve	60.83	6.0	- Mills, et al. (1971).[78]<br>- Frank, et al. (1981).[97]<br>- Hall, et al. (1991).[85]<br>- Hall. (2001).[86]<br>- DeVincenzo, et al. (2010).[80]<br>- Okiro, et al. (2010).[98]<br>- Munywoki, et al. (2015)b.[49]
	ν_2	≥ 1 previous RSV infections	91.25	4.0	
- Kinyanjui, et al. (2015).[1]<br>- Pan-Ngum, et al. (2017).[2] (SAI model)<br>- Brand, et al. (2020).[3]<br>- Kinyanjui, et al. (2020).[4] (SAI model)	ν_1	RSV naïve	40.60	9.0	- Hall, et al. (1976).[83]<br>- Waris, et al. (1992).[99]<br>- Okiro, et al. (2010).[98]
	ν_2	≥ 1 previous RSV infections	93.70	3.9	
- Pitzer, et al. (2015).[7]	ν_1	RSV naïve	36.50	10.0	- Hall, Douglas, Geiman. (1976).[84]<br>- Okiro, et al. (2010).[98]
	ν_2	1 previous RSV infection	52.14	7.0	
	ν_3	≥ 2 previous RSV infections	73.00	5.0	
- Yamin, et al. (2016).[8]	ν_1	RSV naïve	14.04	26.0	- Hall, Douglas, Geiman. (1976).[84]<br>- DeVincenzo, et al. (2010).[80]
	ν_2	≥ 1 previous RSV infections	28.08	13.0	
- Hodgson, et al. (2020).[9]	ν_1	RSV naïve	59.25	6.16	- DeVincenzo, et al. (2010).[80]<br>- Okiro, et al. (2020).[98]
	ν_2	1 previous RSV infection	68.10	5.36	
	ν_3	≥ 2 previous RSV infections	82.29	4.23	
**Continued next page**					

URTI – Upper respiratory tract infection; LRTI – Lower respiratory tract infection; SLRTI – Severe lower respiratory tract infection.

[a] The modelling approach taken assumes that the time from infection to recovery is approximately two weeks. Movement from infectious to recovered compartment is not explicitly modelled.

Supplemental Table A.5.5 (continued): Parameterization of the recovery rate (ν) in RSV DTMs.

Model	Symbol	Description	Rate (per year)	Duration (days)	Reference
**Stratification by age**					
- Arguedas, Santana-Cibrian, Velasco-Hernández. (2019).[50]	ν_{0-4}	0 – 4-year-olds	56.68	6.44	- Calibrated values
	ν_{5-19}	1 – 19-year-olds	91.48	3.99	
	ν_{20-59}	20 – 59-year-olds	81.47	4.48	
	$\nu_{\geq 60}$	≥ 60-year-olds	86.90	4.20	
**Stratification by severity of RSV infection**					
- Pan-Ngum, et al. (2017).[2] (BWI model)<br>- Mahikul, et al. (2019).[21]<br>- Kinyanjui, et al. (2020). (BWI model).[4]	ν_A	Asymptomatic	91.25	4.0	- Waris, et al. (1992).[99]<br>- Hall, et al. (1976).[83]<br>- Okiro, et al. (2010).[98]
	ν_U	URTI	91.25	4.0	
	ν_L	LRTI	40.56	9.0	
	ν_S	SLRTI	40.56	9.0	
**Stratification by nutritional status**					
- Paynter. (2016).[54]	ν_W	Well-nourished	73.00	5.0	- James. (1972).[100]<br>- Tomkins. (1981).[101]<br>- Black, Brown, Becker. (1984).[102]<br>- Heymann. (2008).[90]<br>- Okiro, et al. (2010).[98]
	ν_W	Malnourished	56.15	6.5	

URTI – Upper respiratory tract infection; LRTI – Lower respiratory tract infection; SLRTI – Severe lower respiratory tract infection.

[a] The modelling approach taken assumes that the time from infection to recovery is approximately two weeks. Movement from infectious to recovered compartment is not explicitly modelled.

Supplemental Table A.5.6: Parameterization of the immunity waning rate (γ) in RSV DTMs.

Model	Symbol	Description	Rate (per year)	Duration (days)	Reference
**Unstratified (recovery rate applied uniformly to entire population)**					
- Weber, Weber, Milligan. (2001).[10]<br>- Arenas, González-Parra, Moraño. (2009).[59]<br>- Arenas, González-Parra, Jódar. (2010).[60]<br>- Ponciano, Capistrán. (2011).[37]<br>- Aranda-Lozano, González-Parra, Querales. (2013).[35]<br>- Yamin, et al. (2016).[8]<br>- Nugraha, Nuraini. (2017).[24]<br>- Smith, Hogan, Mercer. (2017).[11]<br>- Rosa, Torres. (2018)a.[25]<br>- Rosa, Torres, (2018)b.[26]	γ	Immunity waning rate	1.80	202.8	- Hall, et al. (1991).[85]
- Acedo, et al. (2010).[15]<br>- Acedo, Moraño, Díez-Domingo. (2010).[16]<br>- Corberán-Vallet, Santonja. (2014).[61]<br>- Jornet-Sanz, et al. (2017).[23]	γ	Immunity waning rate	1.83	199.5	- Hall. (2004).[96]
- Paynter, et al. (2014).[53]	γ	Immunity waning rate	5.84	62.5	- Hall, et al. (1991).[85]
- Kinyanjui, et al. (2015).[1]<br>- Morris, et al. (2015).[81]<br>- Pan-Ngum, et al. (2017).[2] (SAI model)<br>- Brand, et al. (2020).[3]<br>- Kinyanjui, et al. (2020).[4] (SAI model)	γ	Immunity waning rate	2.00	182.5	- Scott, et al. (2006).[103]<br>- Agoti, et al. (2012).[104]<br>- Ohuma, et al. (2012).[44]
- Hodgson, et al. (2020).[9]	γ	Immunity waning rate	1.02	358.9	- Hall, et al. (1991).[85]<br>- Scott, et al. (2006).[103]
- Moore, et al. (2014).[18]<br>- Hogan, et al. (2017).[6]	γ	Immunity waning rate	2.13	171.4	- Calibrated value
- Poletti, et al. (2015).[5]	γ	Immunity waning rate	1.83	199.5	- Calibrated value
- Hogan, et al. (2016).[105]<br>- Campbell, Geard, Hogan. (2020).[12]	γ	Immunity waning rate	1.59	229.6	- Calibrated value[a]
**Continued next page.**					

[a] Value is determined by calibration in Hogan, et al. (2016).[105] and is subsequently reused in Campbell, Geard, Hogan. (2020).[12]

Supplemental Table A.5.6 (continued): Parameterization of the immunity waning rate (γ) in RSV DTMs.

Model	Symbol	Description	Rate (per year)	Duration (days)	Reference
**Stratified by age**					
- Paynter. (2016).[54]	$\gamma_{<2}$	< 2-year-olds	5.84	62.5	- Calibrated value
- Arguedas, Santana-Cibrian, Velasco-Hernández. (2019).[50]	γ_{0-4}	0 – 4-year-olds	10.51	34.7	- Calibrated values
	γ_{5-19}	5 – 19-year-olds	5.85	62.4	
	γ_{20-59}	20 – 59-year-olds	2.80	130.2	
	$\gamma_{\geq 60}$	≥ 60-year-olds	2.88	126.9	
- van Boven, et al. (2020).[22]	$\gamma_{<1}$	< 1-year-olds	2.31	158.0	- Calibrated values
	γ_{1-4}	1 – 4-yearolds	0.46	739.5	
	γ_{5-9}	5 – 9-year-olds	0.19	1,921.1	
	γ_{10-19}	10 – 19-year-olds	0.19	1,921.1	
	γ_{20-44}	20 – 44-year-olds	0.16	2,281.3	
	γ_{45-64}	45 – 64-year-olds	0.22	1,659.1	
	$\gamma_{\geq 65}$	≥ 65-year-olds	0.50	730.0	

[a] Value is determined by calibration in Hogan, et al. (2016).[105] and is subsequently reused in Campbell, Geard, Hogan. (2020).[12]

Supplemental Table A.5.7: Parameterization of the social mixing matrix (C).

Model	Reference
**Literature values**	
- Kinyanjui, et al. (2015).[1]<br>- Pan-Ngum, et al. (2017).[2]	- Scott, et al. (2012).[106]<br>- Kiti, et al. (2014).[107]
- Pitzer, et al. (2015).[7]<br>- Yamin, et al. (2016).[8]<br>- Hogan, et al. (2017).[6]<br>- Goldstein, et al. (2018).[19]<br>- Arguedas, Santana-Cibrian, Velasco-Hernández. (2019).[50]<br>- Campbell, Geard, Hogan. (2020).[12]<br>- Kinyanjui, et al. (2020).[4]	- Wallinga, et al. (2006).[108]<br>- Mossong, et al. (2008).[109]
- Mahikul, et al. (2019).[21]	- Meeyai, et al. (2015).[110]
- Hodgson, et al. (2020).[9]	- Mossong, et al. (2008).[109]<br>- van Hoeck, et al. (2013).[111]
- van Boven, et al. (2020).[22]	- van de Kassteele, van Eijkeren, Wallinga. (2017).[112]
**Calibrated values**	
- Kinyanjui, et al. (2015).[1]	- Calibrated values
- Poletti, et al. (2015).[5]	- Calibrated values
- Kombe, et al. (2019).[20]	- Calibrated values
- Brand, et al. (2020).[3]	- Calibrated values

Appendix A.6: Modelling results

Finally, we provide an overview of the major results of RSV DTMs.

Supplemental Table A.6.1: Summary of results of RSV DTMs.

Model	Summary of results
Weber, Weber, Milligan. (2001).[10]	Two models are developed: a *SIRS* model and an *M-SEIRS4* model. Both models are able to reproduce RSV hospitalization data in four locations: (a) Turku, Finland (which exhibits a biennial pattern), (b) Florida, USA, (c) The Gambia, and (d) Singapore.
White, et al. (2005).[36]	A non-standard model is developed that models RSV groups A and B separately. The model reproduces RSV epidemic data overall, and RSV A and B separately, in two locations: (a) Turku, Finland, and (b) England & Wales, United Kingdom. Following RSV infection, susceptibility of individuals to subsequent homologous or heterologous reinfections is reduced by a factor of 0.36 or 0.84, respectively.
White, et al. (2007).[31]	A system of eight nested models is developed (incl. *SIS*, *SIR*, *SIRS* type models, among others). A model with lifelong partial immunity (i.e., previously infected individuals are less susceptible and less infectious, and are infectious for a shorter duration) was found to best fit RSV epidemic data from nine locations: (a) Porto Alegre, Brazil, (b) Rio de Janeiro, Brazil, (c) England & Wales, United Kingdom, (d) West Midlands, United Kingdom, (e) Finland, (f) Florida, United States, (g) The Gambia, (h) Madrid, (i) Spain, and (j) Singapore.
Arenas, González, Jódar. (2008).[94]	An analysis of the nested models proposed in White, et al. (2007).[31] is performed and conditions for the existence of periodic solutions are established.
Arenas, González-Parra, Moraño. (2009).[59]	Two SDE models analogous to the *SIRS* model of Weber, Weber, Milligan. (2001).[10] are developed: one where the average transmission coefficient (b_0) is specified as a Wiener process, and one where or the birth rate (μ) is specified as a Wiener process. The model reproduces RSV hospitalization data in Valencia, Spain. Analysis of the SDE models finds that the *SIRS* model of Weber, Weber, Milligan. (2001).[10] is more sensitive to stochastic perturbations of average transmission coefficient than it is to stochastic perturbations of birth rate.
Acedo, et al. (2010).[15]	An age-stratified *SIRS* model with vaccination of newborns at birth is developed, accompanied by a cost effectiveness analysis that includes hospitalization, vaccination, and caregiver productivity loss costs. The model is calibrated to data from Valencia, Spain. Higher levels of productivity loss vaccination are associated with a reduction in total costs.
Acedo, Moraño, Díez-Domingo. (2010).[16]	An ABM is developed that is analogous to the *SIRS* model presented in Acedo, et al. (2010).[15]. Individuals are implemented as nodes on a complete graph. A cost effectiveness analysis that includes hospitalization, vaccination, and caregiver productivity loss costs is performed. As with Acedo, et al. (2010).[15], it is found that for higher levels of productivity loss vaccination may result in a reduction in total costs.
**Continued next page.**	

Supplemental Table A.6.1 (continued): Summary of results of RSV DTMs.

Model	Summary of results
Arenas, González-Parra, Jódar. (2010).[60]	A sensitivity analysis is performed on the *SIRS* model of Weber, Weber, Milligan. (2001).[10] calibrated to data from Valencia, Spain. Parameters that are varied include: intial conditions of infectious (I) and recovered (R) compartments, average transmission coefficient (b_0), and birth rate (μ). The model is most sensitive to uncertainties in average transmission. The model is least sensitive to uncertainties in initial conditions.
Leecaster, et al. (2011).[17]	An *SEIR* model was developed for modeling a single season of RSV. The average transmission coefficient (b_0) was found to be correlated with the epidemic start time; together, these quantities are found to explain variation in seasonal epidemic size.
Ponciano, Capistrán. (2011).[37]	An *SIRS* model is modified by changing the incidence rate function from the standard bilinear incidence rate ($\beta\ IS/N$) to Liu-Hethcote-van den Driessche (LHD) incidence rate function ($\beta\ I^2S/\ (I+\alpha)/N$). The model is applied to RSV epidemics from Turku, Finland, and The Gambia using the parameterization and calibration data from Weber, Weber, Milligan. (2001).[10] Inclusion of the LHD incidence rate function results in the disease-free equilibrium always being a local attractor. Comparison of standard and LHD *SIRS* models using Akaike and Bayesian information criteria are favorable to the LHD *SIRS* model.
Mwambi, et al. (2011).[41]	A generalized linear modelling (GLM) approach was adapted to an *SIS* RSV DTM to estimate time-varying disease parameters, e.g. the force of infection. For RSV epidemic data from Kilifi, Kenya, it is found that force of infection peaks in May and January-February.
Aranda-Lozano, González-Parra, Querales. (2013).[35]	The *SIRS* model of Weber, Weber, Milligan. (2001).[10] reproduces RSV detection data from Bogota, Colombia.
Corberán-Vallet, Santonja. (2014).[61]	A *SIRS* stochastic difference equation model was developed where the number of new infected individuals is a binomial random variable with success probability that depends on (a) the number of infected individuals in the previous time step and (b) a time-varying stochastic transmission coefficient. A Bayesian analysis of the model allows for the estimation of the posterior distribution of model parameters and outputs by calibrating to Valencia, Spain.
Moore, et al. (2014).[18]	An age stratified *SEIRS* model is developed that reproduces the biennial epidemic pattern observed in data from Western Australia.
Paynter, et al. (2014).[53]	An *SEIRS2* model is developed for Bohol, Phillipines. The peak in transmissibility of RSV is estimated to occur 49-67 days prior to the peak in RSV detections. Nutritional status and rainfall were identified as two potential seasonal drivers of RSV infection dynamics. Specifically, the peak in transmission ($\beta(t)$) achieves its maximum intensity approximately 7 weeks prior to peak RSV detections and its minimum intensity approximately 19 weeks following peak RSV detections. This is compared to mean birth weight (a proxy for nutrition), which achieves its minimum approximately 10 weeks prior to the peak in RSV detections, and the number of days per week with more than 5mm of precipitation (a proxy for rainfall), which achieves its minimum approximately 17-18 weeks following peak RSV detections.
Kinyanjui, et al. (2015).[1]	An age-structured *M-SIRS3* model incorporating vaccination is developed. The model is calibrated to data from Kilifi, Kenya. The model predicts that, with respect to reduction of disease burden in < 6-month-olds, the optimal age for vaccination is between 5 and 10 months; vaccination of these age cohorts results in a significant reduction in disease in young infants through herd immunity.
Morris, et al. (2015).[81]	The sensitivity of RSV epidemics to birth rates is not captured by the *SIRS* model. The authors implement an *SIRS2* model and find that by including two levels of partial immunity (RSV naïve and at least one previous RSV infection) is sufficient capture sensitivity of RSV epidemics to birth rate.
Pitzer, et al. (2015).[7]	An *M-SIS4* model is calibrated to RSV epidemic data from multiple US states. Correlation was observed between estimated model parameters and climactic variables of temperature, vapor pressure, precipitation, and potential evapotranspiration (PET). Specifically, the amplitude of seasonal fluctuations in the transmission rate (b_1) and the phase shift of the transmission rate (ϕ) were found to be negatively correlated with mean precipitation and mean vapor pressure, and positively correlated with the amplitude and timing of PET.
Poletti, et al. (2015).[5]	An agent-based transmission model is developed that differentiates interactions based on three types of interaction: household, school, and general. The model is calibrated to data from Kilifi, Kenya. It is found that, of the infant infections that occur due to household interactions (39%), a majority (55%) are caused by school-aged children. For the purposes of reducing infant RSV infections, it is found that vaccination of school-age children is nearly as effective as vaccination of infants.
Hogan, et al. (2016).[27]	An age stratified *SEIRS* model is developed for Western Australia. Parameter and bifurcation analyses are provided. Parameter analysis finds that (a) biennial cycles result when b_1 is large, (b) biennial cycles exhibit a delay for intermediate values of μ, and (c) annual cycles predominate when the duration of immunity ($1/\gamma$) is short. Bifurcation analysis confirms the existence of period doubling and period halving bifurcations.
Paynter. (2016).[54]	An *SEIRS* model for children is stratified by nutritional status (well-nourished versus malnourished). Effects of malnutrition on development of severe RSV disease were considered in three scenarios: increased likelihood of infected malnourished children developing severe RSV disease, increased susceptibility of malnourished children in becoming infected, and increased infectiousness of infected malnourished children. The population attributable fraction (PAF) calculated using the model is (a) equal to conventionally calculated PAF for scenarios that did not affect disease transmission and (b) greater than the conventionally calculated PAF for scenarios that did affect disease transmission.
**Continued next page.**	

Supplemental Table A.6.1 (continued): Summary of results of RSV DTMs.

Model	Summary of results
Reis, Shaman. (2016).[68]<br>Reis, Shaman. (2018).[69]	An *SIR* model is developed to model a single season of RSV with the goal of forecasting RSV dynamics, e.g., the epidemic peak. All model parameters are calibrated by iteratively applying an ensemble adjustment Kalman filter (EAKF) to US RSV detection data. Forecasts produced from data up to four weeks prior to the peak in RSV detections produced forecasts that were within 25% of the actual peak magnitude approximately 70% of the time.
Yamin, et al. (2016).[8]	A non-standard age stratified ODE model that includes asymptomatic individuals and vaccination is developed. The model is calibrated to data from four US states: California, Colorado, Pennsylvania, and Texas. Vaccination of < 5-year-olds is the most effective strategy to reduce RSV disease burden in all age strata.
Hogan, et al. (2017).[6]	An age-stratified *M-SEIRS* model with maternal vaccination is developed. Maternal immunization may significantly reduce RSV hospitalizations in infants aged < 6 months.
Jornet-Sanz, et al. (2017).[23]	An extension to Corberán-Vallet, Santonja. (2014).[61] is developed that allows for vaccination of newborns at birth.
Nugraha, Nuraini. (2017).[24]	The *SIRS* model of Weber, Weber, Milligan. (2001).[10] is modified for intervention by vaccination and public awareness campaign. The model is calibrated to data from North Carolina, United States. A combination of vaccination and public awareness campaign result in the greatest reduction in disease burden. The relative contribution of vaccination to reduction in disease burden is greater than that of the public awareness campaign.
Pan-Ngum, et al. (2017).[2]	Qualitatively similar results are reported for two model structures: *M-SIRS3* (see Kinyanjui, et al. (2015).[1]) and ``BWI'' (non-standard model structure). The models are calibrated to data from Kilifi, Kenya. Multiple intervention strategies, i.e., both maternal and infant vaccination, are implemented. For both models (a) vaccination of pregnant women is less effective in reducing disease burden in < 5-year-olds than vaccination of infants, and (b) the herd immunity effect is strongest for vaccines that reduce infectiousness and duration of infectiousness.
Smith, Hogan, Mercer. (2017).[11]	The *SIRS* model of Weber, Weber, Milligan. (2001).[10] is extended by adding maternal vaccination or vaccination at discrete time points. Simulation demonstrates that the disease-free equilibrium of the model with vaccination at discrete time points can be destabilized under extreme conditions, e.g., 100% coverage with a vaccine that confers 10,000 times increased infectiousness.
Goldstein, et al. (2018).[19]	The authors present United States RSV hospitalization data stratified by age and compute the relative risk (RR) for each age strata, i.e., the ratio of normalized before peak counts to normalized after peak counts for each age strata. The RR is found to be highest for children 3 – 4 and 5 – 6-year-olds in 5 out of 11 seasons, and is generally higher in 1 – 10-year-olds versus either < 1-year-olds or > 10-year-olds. An *SIR* mathematical model was developed to validate these results and to simulate the effect of vaccination of different age strata. Vaccination of age groups with higher RR values was most effective in reducing RSV infections.
Rosa, Torres. (2018)a.[25]	*SIRS* and *SEIRS* models are extended to allow for treatment of infectious individuals. The models were calibrated to RSV epidemic data from Florida, United States. A system of equations is derived for the optimal control function $T(t)$ by using the Pontryagin maximum principle, where $T(t)$ is a function that determines the intensity of treatment program for infectious individuals.
Rosa, Torres. (2018)b.[26]	An extension to Rosa, Torres. (2018)a.[25] in which systems of fractional differential equations are developed that are analogous to *SIRS* and *SEIRS* compartmental models. Fractional order of differentiation is estimated by fitting to RSV hospitalization data from Florida, United States. A system of equations is derived for the optimal control function $T(t)$ by using the Pontryagin maximum principle.
Arguedas, Santana-Cibrian, Velasco-Hernández. (2019).[50]	An age stratified *SEIRS* model is developed with four age strata: 0 – 4-year-olds, 5 – 19-year-olds, 20 – 59-year-olds, and ≥ 60-year-olds. The model is calibrated to age stratified data from Luis Potosí, Mexico, and the roles played by different age strata in the epidemic dynamics are inferred from parameter estimates. Children (< 5-year-olds) are (a) more likely to get sick, (b) remain infectious longer, and (c) lose temporary immunity to reinfection faster than other age strata. It is concluded that young children are the primary contributors to the spread of RSV.
Baker, et al. (2019).[51]	A time series implementation of an *SIR* model is developed, resulting in estimates for the transmission parameter as a function of time for much of the United States and Mexico. An inverse relationship between humidity and log transmission and a linear relationship between rainfall and transmission are observed. Effects of climate change on RSV infection dynamics are considered through simulation.
Kombe, et al. (2019).[20]	An agent-based transmission model was developed for household dynamics of RSV A and RSV B epidemics. The model is calibrated to data from Kilifi, Kenya. Following RSV infection, susceptibility of individuals to subsequent homologous or heterologous reinfection is reduced by 47% or 39%, respectively. The rate of pairwise transmission is lower in larger households (> 7 members), but overall household transmission rate is higher in larger households. Between 32-53% of RSV transmissions are attributed to within household interactions.
Mahikul, et al. (2019).[21]	An extension of the BWI model first proposed in Pan-Ngum, et al. (2017).[2] was developed to incorporate population and household structure. The model is calibrated to data from Thailand. Extended families (i.e., three generations living together) are majority contributors to the force of infection, and their contribution is expected to increase in the near future.
**Continued next page.**	

Supplemental Table A.6.1 (continued): Summary of results of RSV DTMs.

**Model**	**Summary of results**
Brand, et al. (2020).[3]	A non-standard *SIRS* model implementing household structure was developed to investigate the effects of maternal vaccination (conferring protection to both mother and child) and vaccination of the entire household at time of birth. The model is calibrated to data from Kilifi, Kenya. A significant reduction in hospitalizations can be achieved by vaccination a relatively small subset of the population, i.e., vaccination coverage of 75% for maternal and household vaccinations at time of birth results in a 50% reduction in RSV hospitalizations (assuming maternal vaccination increases duration of natural maternal immunity by 75 days).
Campbell, Geard, Hogan. (2020).[12]	An *M-SEIRS* ABM implementing household structure was developed to investigate the effects of maternal vaccination (conferring protection to both mother and child). The model is calibrated to data from Western Australia (Perth, Australia). At 70% coverage the reduction in infections for < 3- and 3 – 6-month-olds was 16.6% and 5.3%, respectively; there was some evidence of infections being delayed from the first to second year of life.
Hodgson, et al. (2020).[9]	An age stratified M-SEIRS3 ODE model is adapted to include asymptomatic individuals, i.e., Exposed individuals become infectious and symptomatic (I) or infectious and asymptomatic (A). The effects of palivizumab immunoprophylaxis, long-acting monoclonal antibody immunoprophylaxis, maternal vaccination, and vaccination are investigated. The model is calibrated to data from England, UK. A cost-effectiveness analysis is performed. The maximum cost-effective purchase price for long-acting monoclonal antibody immunoprophylaxis administered to all infants is £90 when compared against current palivizumab immunoprophylaxis. For maternal vaccination the maximum cost-effective purchase price £85. For vaccinating 2-month-old infants the maximum cost-effective purchase price is £95. Vaccination of pre-school and school-age children were not cost-effective relative to vaccination of older adults. Vaccination of older adults (≥ 75-year-olds) is £21.
Kinyanjui, et al. (2020).[4]	Models presented previously in Pan-Ngum, et al. (2017).[2] were calibrated to data from the United Kingdom. Vaccination of infants is implemented for multiple vaccines with properties varying by dosing schedule and reduction in risk of primary infection, duration of infectiousness, infectiousness, and risk of upper, lower, and severe lower respiratory tract infections. The greatest reductions in disease burden for < 5-year-olds result from vaccines that reduce infectiousness and duration of infectiousness.
Seroussi, Levy, Yom-Tov. (2020).[14]	A multi-compartment *SIR* model was calibrated to United States Internet data, i.e., Google searches for the term ``RSV" stratified by US state. Inter-state infection rates are correlated ($\rho = 0.30$) with human mobility data harvested from Twitter. Model paramters are found to be relatively constant year-to-year. The model is able to predict infection rates and timing of infection peaks in each state for the current season using (a) the first seven weeks of RSV data and (b) the previous year's parameter values.
van Boven, et al. (2020).[22]	An age stratified *SIR* model is adapted to model RSV epidemics by coupling it to a discrete mapping function that maps the system at the end of one epidemic to the initial conditions of the subsequent epidemic. Maternal and infant (< 6-month-olds) vaccination are investigated. Maternal vaccination decreased attack rate in < 1-year-olds by 26%, but increased the attack rate in 1 – 4-year-olds and 5 – 9-year-olds by 12.5% and 3.5%, respectively. Infant vaccination decreases the attack rate in < 1-year-olds, 1 – 4-year-olds and 5 – 9-year-olds by 29.8%, 20.8% and 8.2%, respectively.

References

1. Kinyanjui TM, House TA, Kiti MC, Cane PA, Nokes DJ, Medley GF. Vaccine Induced Herd Immunity for Control of Respiratory Syncytial Virus Disease in a Low-Income Country Setting. *PLOS ONE* 2015; **10**(9): e0138018.

2. Pan-Ngum W, Kinyanjui T, Kiti M, et al. Predicting the relative impacts of maternal and neonatal respiratory syncytial virus (RSV) vaccine target prodcut profiles: A consensus modelling approach. *Vaccine* 2017; **35**: 403-9.

3. Brand S, Munywoki PK, Walumbe D, Keeling M, Nokes DJ. Reducing RSV hospitalization in a lower-income country by vaccinating mothers-to-be and their households. *eLife* 2020; **9**: e47003.

4. Kinyanjui T, Pan-Ngum W, Saralamba S, Taylor S, White LJ, Nokes DJ. Model evaluation of target product profiles of an infant vaccine against respiratory syncytial virus (RSV) in a developed country setting. *Vaccine: X* 2020; **4**: 100055.

5. Poletti PM, S., Ajelli M, Manfredi P, Munywoki PK, Nokes DJ, Melegaro A. Evaluating vaccination strategies for reducing infant respiratory syncytial virus infection in low-income settings. *BMC Med* 2015; **13**: 49-59.

6. Hogan AB, Campbell PT, Blyth CC, et al. Potential impact of a maternal vaccine for RSV: A mathematical modelling study. *Vaccine* 2017; **35**: 6172-9.

7. Pitzer VE, Viboud C, Alonso WJ, et al. Environmental Drivers of the Spatiotemporal Dynamics of Respiratory Syncytial Virus in the United States. *PLoS Pathogens* 2015; **11**(1): e1004591.

8. Yamin D, Jones FK, DeVincenzo JP, et al. Vaccination strategies against respiratory syncytial virus. *Proceedings of the National Academy of Sciences of the United States of America* 2016; **113**(46): 13239-44.

9. Hodgson D, Pebody R, Panovska-Griffiths J, Baguelin M, Atkins KE. Evaluating the next generation of RSV intervention strategies: a mathematical modelling study and cost-effectiveness analysis. *BMC Med* 2020; **18**(1): 348.

10. Weber A, Weber M, Milligan P. Modeling epidemics caused by respiratory syncytial virus (RSV). *Math Biosci* 2001; **172**: 95-113.

11. Smith R, Hogan AB, Mercer GN. Unexpected Infection Spikes in a Model of Respiratory Syncytial Virus Vaccination. *Vaccines* 2017; **5**(2): 1-15.

12. Campbell PT, Geard N, Hogan AB. Modelling the household-level impact of a maternal respiratory syncytial virus (RSV) vaccine in a high-income setting. *BMC Med* 2020; **18**(1): 319.

13. Glezen WP, Taber LH, Frank AL, Kasel JA. Risk of primary infection and reinfection with respiratory syncytial virus. *Am J Dis Child* 1986; **140**(6): 543.

14. Seroussi I, Levy N, Yom-Tov E. Multi-season analysis reveals the spatial structure of disease spread. *Physica A* 2020; **547**: 124425.

15. Acedo L, Diez-Domingo J, Morano J-A, Villanueva R-J. Mathematical modelling of respiratory syncytial virus (RSV): vaccination strategies and budget applications. *Epidemiol Infect* 2010; **138**(8): 853-60.

16. Acedo L, Moraño J-A, Díez-Domingo J. Cost analysis of a vaccination strategy for respiratory syncytial virus (RSV) in a network model. *Math Comput Model* 2010; **52**(7): 1016-22.

17. Leecaster M, Gesteland P, Greene T, et al. Modeling the variations in pediatric respiratory syncytial virus seasonal epidemics. *Bmc Infect Dis* 2011; **11**.

18. Moore HC, Jacoby P, Hogan AB, Blyth CC, Mercer GN. Modelling the Seasonal Epidemics of Respiratory Syncytial Virus in Young Children. *PLOS ONE* 2014; **9**(6): e100422.

19. Goldstein E, Nguyen HH, Liu P, et al. On the Relative Role of Different Age Groups During Epidemics Associated With Respiratory Syncytial Virus. *J Infect Dis* 2018; **217**: 238-44.

20. Kombe IK, Munywoki PK, Baguelin M, Nokes DJ, Medley GF. Model-based estimates of transmission of respiratory syncytial virus within households. *Epidemics* 2019; **27**: 1-11.

21. Mahikul W, White LJ, Poovorawan K, et al. Modeling household dynamics on Respiratory Syncytial Virus (RSV). *PLOS One* 2019; **14**(7): e0219323.

22. van Boven M, Teirlinck AC, Meijer A, et al. Estimating Transmission Parameters for Respiratory Syncytial Virus and Predicting the Impact of Maternal and Pediatric Vaccination. *J Infect Dis* 2020; **222**(Supplement_7): S688-s94.

23. Jornet-Sanz M, Corberan-Vallet A, Santonja FJ, Villanueva RJ. A Bayesian stochastic SIRS model with a vaccination strategy for the analysis of respiratory syncytial virus. *SORT-Stat Oper Res T* 2017; **41**(1): 159-76.

24. Nugraha ES, Nuraini N. Simple Vaccination and Prevention Model of Respiratory Syncytial Virus. *Far East J Math Sci* 2017; **102**(9): 1865-80.

25. Rosa S, Torres DFM. Parameter Estimation, Sensitivity Analysis and Optimal Control of a Periodic Epidemic Model with Application to HRSV in Florida. *Stat Optim Inf Comput* 2018; **6**: 139-49.

26. Rosa S, Torres DFM. Optimal control of a fractional order epidemic model with application to human respiratory syncytial virus infection. *Chaos, Solitons and Fractals* 2018; **117**: 142-9.

27. Hogan AB, Glass K, Moore HC, Anderssen RS. Exploring the dynamics of respiratory syncytial virus (RSV) transmission in children. *Theor Popul Biol* 2016; **110**: 78-85.

28. Hall CB. Respiratory syncytial virus. In: Feigin RD, Cherry JD, eds. Textbook of Paediatric Infectious Diseases (Volume II). 1 ed: WB Saunders Company, Philadelphia; 1981.

29. Hogan AB, Anderssen RS, Davis S, et al. Time series analysis of RSV and bronchiolitis seasonality in temperate and tropical Western Australia. *Epidemics* 2016; **16**: 49-55.

30. Jacoby P, Glass K, Moore HC. Characterizing the risk of respiratory syncytial virus in infants with older siblings: a population-based birth cohort study. *Epidemiol Infect* 2017; **145**: 266-71.

31. White LJ, Mandl JN, Gomes MGM, et al. Understanding the transmission dynamics of respiratory syncytial virus using multiple time series and nested models. *Math Biosci* 2007; **209**: 222-39.

32. Straliotto SM, Nestor SM, Siqueira MM. Respiratory syncytial virus groups A and B in Porto Alegre, Brazil, from 1990 to 1995 and 1998. *J Memórias do Instituto Oswaldo Cruz* 2001; **96**: 155-8.

33. Siqueira MM, Nascimento JP, Anderson LJ. Antigenic characterization of respiratory syncytial virus group A and B isolates in Rio de Janeiro, Brazil. *J Clin Microbiol* 1991; **29**(3): 557-9.

34. Nascimento JP, Siqueira MM, Sutmoller F, et al. Longitudinal study of acute respiratory diseases in Rio de Janeiro: occurrence of respiratory viruses during four consecutive years. *J Revista do Instituto de Medicina Tropical de São Paulo* 1991; **33**: 287-96.

35. Aranda-Lozano D, González-Parra G, Querales J. Modelamiento de la transmisión del Virus Respiratorio Sincitial (VRS) en niños menores de cinco años. *Rev Salud Pública* 2013; **15**(4): 637-47.

36. White LJ, Waris M, Cane PA, Nokes DJ, Medley GF. The transmission dynamics of groups A and B human respiratory syncytial virus (hRSV) in England & Wales and Finalnd: seasonality and cross-protection. *Epidemiol Infect* 2005; **133**: 279-89.

37. Ponciano JM, Capistrán MA. First principles modeling of nonlinear incidence rates in seasonal epidemics. *PLoS Comput Biol* 2011; **7**(2).

38. Waris M. Pattern of respiratory syncytial virus epidemics in Finland: two-year cycles with alternating prevalence of groups A and B. *J Infect Dis* 1991; **163**: 464.

39. Weber MW, Dackour R, Usen S, et al. The clinical spectrum of respiratory syncytial virus disease in The Gambia. *Pediatr Infect Dis J* 1998; **17**(3): 224-30.

40. Cane PA, Weber M, Sanneh M, Dackour R, Pringle CR, Whittle H. Molecular epidemiology of respiratory syncytial virus in The Gambia. *Epidemiol Infect* 1999; **122**(1): 155-60.

41. Mwambi H, Ramroop S, White LJ, et al. A frequentist approach to estimating the force of infection for a respiratory disease using repeated measurement data from a birth cohort. *Statistical Methods in Medical Research* 2011; **20**(5): 551-70.

42. Nokes DJ, Okiro EA, Ngama M, et al. Respiratory syncytial virus epidemiology in a birth cohort from Kilifi district, Kenya: infection during the first year of life. *J Infect Dis* 2004; **190**(10): 1828-32.

43. Nokes DJ, Okiro EA, Ngama M, et al. Respiratory Syncytial Virus Infection and Disease in Infants and Young Children Observed from Birth in Kilifi District, Kenya. *Clin Infect Dis* 2008; **46**: 50-7.

44. Ohuma EO, Okiro EA, Ochola R, et al. The natural history of respiratory syncytial virus in a birth cohort: the influence of age and previous infection on reinfection and disease. *Am J Epidemiol* 2012; **176**: 794-802.

45. Nokes DJ, Ngama M, Bett A, et al. Incidence and severity of respiratory syncytial virus pneumonia in rural Kenyan children identified through hospital surveillance. *Clin Infect Dis* 2009; **49**(9): 1341-9.

46. Munywoki PK. Transmission of Respiratory Syncytial Virus in Households: Who Acquires Infection From Whom. *PhD Thesis, The Open University, UK* 2013.

47. Munywoki PK, Koech DC, Agoti CN, Lewa C, Cane PA, Medley GF. The source of respiratory syncytial virus infection in infants: a household cohort study in rural Kenya. *J Infect Dis* 2014; **209**(11): 1685-92.

48. Munywoki PK, Koech DC, Agoti CN, et al. Frequent Asymptomatic Respiratory Syncitial Virus Infections During an Epidemic in a Rural Kenyan Household Cohort. *J Infect Dis* 2015; **212**: 1711-8.

49. Munywoki PK, Koech DC, Agoti CN, et al. Influence of age, severity of infection, and co-infection on the duration of respiratory syncytial virus (RSV) shedding. *Epidemiol Infect* 2015; **143**(4): 804-12.

50. Arguedas Y, Santana-Cibrian M, Velasco-Hernández. Transmission dynamics of acute respiratory diseases in a population structured by age. *Math Biosci Eng* 2019; **16**: 7477.

51. Baker R, Mahmud A, Wagner C, et al. Epidemic dynamics of respiratory syncytial virus in current and future climates. *Nat Commun* 2019; **10**: 5512.

52. Vos LM, Teirlinck AC, Lozano JE, et al. Use of the moving epidemic method (MEM) to assess national surveillance data for respiratory syncytial virus (RSV) in the Netherlands, 2005 to 2017. *Eurosurveillance* 2019; **24**(20): 1800469.

53. Paynter S, Yakob L, Simoes EAF, et al. Using Mathematical Transmission Modelling to Investigate Drivers of Respiratory Syncytial Virus Seasonality in Children in the Philippines. *PLOS ONE* 2014; **9**(2): e90094.

54. Paynter S. Incorporating Transmission into Causal Models of Infectious Diseases for Improved Understanding of the Effect and Impact of Risk Factors. *American Journal of Epidemiology* 2016; **183**(6): 574-82.

55. Lucero MG, Nohynek H, Williams G, et al. Efficacy of an 11-Valent Pneumococcal Conjugate Vaccine Against Radiologically Confirmed Pneumonia Among Children Less Than 2 Years of Age in the Philippines: A Randomized, Double-Blind, Placebo-Controlled Trial. *Pediatr Infect Dis J* 2009; **28**(6).

56. Simões EAF, Patel C, Sung W-K, et al. Pathogen Chip for Respiratory Tract Infections. *J Clin Microbiol* 2013; **51**(3): 945-53.

57. Chew F, Doraisingham S, Ling A, Kumarasinghe G, Lee B. Seasonal trends of viral respiratory tract infections in the tropics. *Epidemiol Infect* 1998; **121**: 121.

58. García García ML, Ordobás Gabin M, Calvo Rey C, et al. Infecciones virales de vías respiratorias inferiores en lactantes hospitalizados: etiología, características clínicas y factores de riesgo. *Anales de Pediatría* 2001; **55**(2): 101-7.

59. Arenas AJ, Gonzalez-Parray G, Morano J-A. Stochastic modeling of the transmission of respiratory syncytial virus (RSV) in the region of Valencia, Spain. *BioSystems* 2009; **96**: 206-12.

60. Arenas AJ, González-Parra G, Jódar L. Randomness in a mathematical model for the transmission of respiratory syncytial virus (RSV). *Math Comput Simulat* 2010; **80**(5): 971-81.

61. Corberán-Vallet A, Santonja FJ. A Bayesian SIRS model for the analysis of respiratory syncytial virus in the region of Valencia, Spain. *Biom J* 2014; **56**(5): 808-18.

62. Fry AM, Chittaganpitch M, Baggett HC, et al. The burden of hospitalized lower respiratory tract infection due to respiratory syncytial virus in rural Thailand. *PLoS ONE* 2010; **5**(11): e15098.

63. Naorat S, Chittaganpitch M, Thamthitiwat S, et al. Hospitalizations for acute lower respiratory tract infection due to respiratory syncytial virus in Thailand, 2008-2011. *J Infect Dis* 2013; **208 Suppl 3**: S238-45.

64. Cane P, Matthews D, Pringle C. Analysis of respiratory syncytial virus strain variation in successive epidemics in one city. *J Clin Microbiol* 1994; **32**: 1-4.

65. Zhao H, Green H, Lackenby A, et al. A new laboratory-based surveillance system (Respiratory DataMart System) for influenza and other respiratory viruses in England: results and experience from 2009 to 2012. *Eurosurveillance* 2014; **19**(3): 20680.

66. Halstead DC, Jenkins SG. Continuous non-seasonal epidemic of respiratory syncytial virus infection in the southeast United States. *South Med J* 1998; **91**(5): 433-6.

67. Wilfret DA, Baker BT, Palavecino E, Moran C, Benjamin DK, Jr. Epidemiology of respiratory syncytial virus in various regions within North Carolina during multiple seasons. *N C Med J* 2008; **69**(6): 447-52.

68. Reis J, Shaman J. Retrospective Parameter Estimation and Forecast of Respiratory Syncytial Virus in the United States. *PLoS Comput Biol* 2016; **12**(10): e1005133.

69. Reis J, Shaman J. Simulation of four respiratory viruses and inference of epidemiological parameters. *Infect Dis Model* 2018; **3**: 23-34.

70. Oren E, Frere J, Yom-Tov E, Yom-Tov E. Respiratory syncytial virus tracking using internet search engine data. *BMC Public Health* 2018; **18**(1): 445.

71. Ogilvie MM, Vathenen AS, Radford M, Codd J, Key S. Maternal antibody and respiratory syncytial virus infection in infancy. *J Med Virol* 1981; **7**(4): 263-71.

72. Ochola R, Sande C, Fegan G, et al. The Level and Duration of RSV-Specific Maternal IgG in Infants in Kilifi Kenya. *PLoS ONE* 2009; **4**(12): e8088.

73. Glezen WP, Paredes A, Allison JE, Taber LH, Frank AL. Risk of respiratory syncyital virus infection for infants from low-income families in relationship to age, sex, ethnic group, and maternal antibody level. *J Pediatr* 1981; **98**(5): 708.

74. Henderson FW, Collier AM, Clyde FW, Denny FW. Respiratory-syncytial-virus infections, reinfections and immunity. A prospective, longitudinal study in young children. *N Engl J Med* 1979; **600**(10): 530.

75. Cox MJ, Azevedo RS, Cane PA, Massad E, Medley GF. Seroepidemiological study of respiratory syncytial virus in Sao Paulo State, Brazil. *J Med Virol* 1998; **55**(3): 234-9.

76. Kapikian AZ, Bell JA, Mastrota FM, Johnson KM, Huebner RJ, Chanock RM. An outbreak of febrile illness and pneumonia associated with respiratory syncytial virus infection. *Am J Epidemiol* 1961; **74**(3): 234-48.

77. Kravetz HM, Knight V, Chanock RM, et al. Respiratory syncytial virus III. Production of illness and clinical observations in adult volunteers. *JAMA* 1961; **176**: 657.

78. Mills J, Van Kirk JE, Wright PF, Chanock RM. Experimental respiratory syncytial virus infection of adults. Possible mechanisms of resistance to infection and illness. *J Immunol* 1971; **107**(1): 123-30.

79. Watt PJ, Robinson BS, Pringle CR, Tyrrel DAJ. Determinants of susceptibility to challenge and the antibody response of adult volunteers given experimental respiratory syncytial virus vaccines. *Vaccine* 1990; **8**(3): 231-6.

80. DeVincenzo JP, Wilkinson T, Vaishnaw A, et al. Viral load drives disease in humans experimentally infected with respiratory syncytial virus. *Am J Respir Crit Care Med* 2010; **182**(10): 1305-14.

81. Morris SE, Pitzer VE, Viboud C, Metcalf CJE, Bjørnstad ON, Grenfell BT. Demographic buffering: Titrating the effects of birth rate and imperfect immunity on epidemic dynamics. *Journal of the Royal Society Interface* 2015; **12**(104).

82. Monto AS, Bryan ER, Rhodes LM. The Tecumseh study of respiratory illness: VII. Further observations on the occurrence of respiratory syncytial virus and mycoplasma pneumoniae infections. *Am J Epidemiol* 1974; **100**(6): 458-68.

83. Hall CB, Geiman JM, Biggar R, Kotok DI, Hogan PM, Douglas JRG. Respiratory syncytial virus infections within families. *N Engl J Med* 1976; **294**(8): 414.

84. Hall CB, Douglas JRG, Geiman JM. Respiratory syncytial virus infections in infants: quantitation and duration of shedding. *J Pediatr* 1976; **89**(1): 11.

85. Hall CB, Walsh EE, Long CE, Schnabel KC. Immunity to and frequency of reinfection with respiratory syncytial virus. *J Infect Dis* 1991; **163**(4): 693.

86. Hall CB, Long CE, Schnabel KC. Respiratory Syncytial Virus Infections in Previously Healthy Working Adults. *Clin Infect Dis* 2001; **33**(6): 792-6.

87. Fairchok MP, Martin ET, Chambers S, et al. Epidemiology of viral respiratory tract infections in a prospective cohort of infants and toddlers attending daycare. *J Clin Virol* 2010; **49**(1): 16-20.

88. Ditchburn RK, McQuillin J, Gardner PS, Court SD. Respiratory syncytial virus in hospital cross-infection. *Br Med J* 1971; **3**(776): 671.

89. Crowcroft N, Zambon M, Harrison T, Mok W, Heath P, Miller E. Respiratory syncytial virus infection in infants admitted to paediatric intensive care units in London, and in their families. *Eur J Pediatr* 2008; **167**: 395-9.

90. Heymann DL. Control of Communicable Diseases Manual. Washington, D.C.: American Public Health Association; 2008.

91. Lessler J, Reigh NG, Brookmeyer R, Perl TM, Nelson KE, Cummings DA. Incubation periods of acute respiratory viral infections: a systematic review. *Lancet Infect Dis* 2009; **9**(5): 291-300.

92. Communicable Disease Control Handbook. 2 ed: Blackwell Publishing; 2005.

93. Lee FE, Walsh EE, Falsey AR, Betts RF, Treanor JJ. Experimental infection of humans with A2 respiratory syncytial virus. *Antiviral Res* 2004; **63**(3): 191-6.

94. Arenas AJ, González G, Jódar L. Existence of periodic solutions in a model of respiratory syncytial virus RSV. *J Math Anal Appl* 2008; **344**: 969-80.

95. Collins P, McIntosh K, Chanock RM. Respiratory syncytial virus. In: Fields B, Knipe D, Howley P, eds. Fields Virology. Philadelphia: Lippincott-Raven Publishers; 1996: 1313.

96. Hall CB. Respiratory syncytial virus and human metapneumovirus. In: Feigin RD, Cherry JD, Demmler GJ, Kaplan SL, eds. Textbook of Pediatric Infectious Diseases. Philadelphia, PA, USA: Saunders; 2004: 2315-41.

97. Frank AL, Taber LH, Wells CR, Wells JM, Glezen WP, Paredes A. Patterns of shedding of myxoviruses and paramyxoviruses in children. *J Infect Dis* 1981; **144**(5): 433-41.

98. Okiro EA, White LJ, Ngama M, Cane PA, Medley GF, Nokes DJ. Duration of shedding of respiratory syncytial virus in a community study of Kenyan children. *Bmc Infect Dis* 2010; **10**(1): 15.

99. Waris M, Meurman O, Mufson MA, Ruuskanen O, Halonen P. Shedding of infectious virus and virus antigen during acute infection with respiratory syncytial virus. *J Med Virol* 1992; **38**(2): 111-6.

100. James JW. Longitudinal study of the morbidity of diarrheal and respiratory infections in malnourished children. *The American Journal of Clinical Nutrition* 1972; **25**(7): 690-4.

101. Tomkins A. Nutritional status and severity of diarrhoea among pre-school children in rural Nigeria. *Lancet* 1981; **1**(8225): 860-2.

102. Black RE, Brown KH, Becker S. Malnutrition is a determining factor in diarrheal duration, but not incidence, among young children in a longitudinal study in rural Bangladesh. *Am J Clin Nutr* 1984; **39**(1): 87-94.

103. Scott PD, Ochola R, Ngama M, et al. Molecular analysis of respiratory syncytial virus reinfections in infants from coastal Kenya. *J Infect Dis* 2006; **193**(1): 59-67.

104. Agoti CN, Mwihuri AG, Sande CJ, et al. Genetic relatedness of infecting and reinfecting respiratory syncytial virus strains identified in a birth cohort from rural Kenya. *J Infect Dis* 2012; **206**(10): 1532-41.

105. Hogan AB, Glass KlM, H.C., Anderssen RS. Age Structures in Mathematical Models for Infectious Diseases, with a Case Study of Respiratory Syncytial Virus. Applications + Practical Conceptualization + Mathematics = fruitful Innovation. Tokyo: Springer; 2016: 105-16.

106. Scott JAG, Bauni E, Moisi JC, et al. Profile: The Kilifi Health and Demographic Surveillance System (KHDSS). *Int J Epidemiol* 2012; **41**(3): 650-7.

107. Kiti MC, Kinyanjui TM, Koech DC, Munywoki PK, Medley GF, Nokes DJ. Quantifying Age-Related Rates of Social Contact Using Diaries in a Rural Coastal Population of Kenya. *PLoS ONE* 2014; **9**(8): e104786.

108. Wallinga J, Teunis P, Kretzschmar M. Using Data on Social Contacts to Estimate Age-specific Transmission Parameters for Respiratory-spread Infectious Agents. *Am J Epidemiol* 2006; **164**(10): 936-44.

109. Mossong J, Hens N, Jit M, et al. Social Contacts and Mixing Patterns Relevant to the Spread of Infectious Diseases. *PLoS Med* 2008; **5**(3): e74.

110. Meeyai A, Praditsitthikorn N, Kotirum S, et al. Seasonal Influenza Vaccination for Children in Thailand: A Cost-Effectiveness Analysis. *PLoS Med* 2015; **12**(5): e1001829.

111. van Hoek AJ, Andrews N, Campbell H, Amirthalingam G, Edmunds WJ, Miller E. The Social Life of Infants in the Context of Infectious Disease Transmission; Social Contacts and Mixing Patterns of the Very Young. *PLoS ONE* 2013; **8**(10): e76180.

112. van de Kassteele J, van Eijkeren J, Wallinga J. Efficient estimation of age-specific social contact rates between men and women. *Ann Appl Stat* 2017; **11**(1): 320-39.
